# PD-1-Mediated PI3K/Akt/mTOR, Caspase 9/Caspase 3 and ERK Pathways Are Involved in Regulating the Apoptosis and Proliferation of CD4^+^ and CD8^+^ T Cells During BVDV Infection *in vitro*

**DOI:** 10.3389/fimmu.2020.00467

**Published:** 2020-03-17

**Authors:** Yu Liu, Shanshan Liu, Chenhua Wu, Wenjing Huang, Bin Xu, Shuai Lian, Li Wang, Shan Yue, Nannan Chen, Zhanbo Zhu

**Affiliations:** ^1^College of Animal Science and Veterinary Medicine, Heilongjiang Bayi Agricultural University, Daqing, China; ^2^Engineering Research Center of Prevention and Control of Cattle Diseases, Daqing, China; ^3^Heilongjiang Provincial Key Laboratory of Prevention and Control of Bovine Diseases, Daqing, China

**Keywords:** programmed death-1 (PD-1), bovine viral diarrhea virus (BVDV), immune dysfunction, PI3K/Akt/mTOR pathway, lymphocyte

## Abstract

Acute infection of bovine viral diarrhea virus (BVDV) is associated with immune dysfunction and can cause peripheral blood lymphopenia and lymphocyte apoptosis. Our previous study has confirmed that programmed death-1 (PD-1) blockade inhibits peripheral blood lymphocyte (PBL) apoptosis and restores proliferation and anti-viral immune functions of lymphocytes after BVDV infection *in vitro*. However, the immunomodulatory effects of PD-1 pathway on major PBL subsets are unclear and their underlying molecular mechanisms need to be further studied. Therefore, in this study, we examined PD-1 expression in bovine PBL subsets after BVDV infection *in vitro* and analyzed the effects of PD-1 blockade on the apoptosis and proliferation of CD4^+^ and CD8^+^ T cells and expression of PD-1 downstream signaling molecules. The results showed that PD-1 expression was enhanced on CD4^+^ and CD8^+^ T cells, but not on CD21^+^ B cells after cytopathic (CP) BVDV (strain NADL) and non-cytopathic (NCP) BVDV (strain KD) infection *in vitro* and PD-1 blockade significantly reduced the apoptosis of CD4^+^ and CD8^+^ T cells after these two strains infection. Remarkably, PD-1 blockade significantly increased the proliferation of CD4^+^ and CD8^+^ T cells after CP BVDV infection, but only significantly increased the proliferation of CD4^+^ T cells after NCP BVDV infection. In addition, we confirmed that PD-1-mediated PI3K/Akt/mTOR, caspase 9/caspase 3 and ERK pathways are involved in regulating the apoptosis and proliferation of CD4^+^ and CD8^+^ T cells during BVDV infection *in vitro*. Notably, ERK is involved in the regulation mechanism PD-1 mediated only when the cells are infected with CP BVDV. Our findings provide a scientific basis for exploring the molecular mechanism of immune dysfunction caused by acute BVDV infection.

## Introduction

Bovine viral diarrhea virus (BVDV) is a significant cause of major economic losses in cattle worldwide and a potentiator for other diseases (1), including both cytopathic (CP) and non-cytopathic (NCP) biotypes (2). Acute BVDV infection is associated with immune dysfunction and can cause peripheral blood lymphopenia and apoptosis (3). There is huge difference in lymphopenia between strains (4–6). However, the molecular mechanism of the immune dysfunction is not entirely clear.

The programmed death-1 (PD-1) pathway plays a critical role in the regulation of immune response and the maintenance of lymphocyte homeostasis (7, 8). Many viruses, such as human immunodeficiency virus (HIV) (9), hepatitis C virus (HCV) (10) and bovine leukemia virus (BLV) (11), can cause lymphocyte apoptosis, inhibit lymphocyte proliferation and induce functional exhaustion of lymphocytes via the PD-1 pathway. More notably, blocking PD-1 pathway improves T cell activation and proliferation and restores T cell function and antiviral immunity (12, 13). To reveal the role of PD-1 in peripheral blood lymphopenia, apoptosis and immune dysfunction after acute BVDV infection, we have investigated the effect of BVDV infection on the expression of PD-1 and PD-L1 on peripheral blood mononuclear cells (PBMC) and analyzed the effect of PD-1 pathway on peripheral blood lymphocyte (PBL) immune functions by PD-1 blockade (14). We found that PD-1 and PD-L1 upregulation after CP and NCP BVDV infection was accompanied with decreased proliferation and increased apoptosis of PBL *in vitro* (14). Remarkably, PD-1 blockade inhibits PBL apoptosis and restores proliferation and anti-viral immune functions of PBL.

Nevertheless, the immunomodulatory effect of the PD-1 pathway on major PBL subsets, such as T helper cells, cytotoxic T cells and B cells in acute BVDV infection is still unclear. Moreover, the molecular mechanism underlying this immunomodulatory effect needs to be further studied. Therefore, in this study, we examined PD-1 expression on bovine PBL subsets after BVDV infection *in vitro* and analyzed the effects of PD-1 blockade on the apoptosis and proliferation of CD4^+^ and CD8^+^ T cells and the expression of PD-1 downstream signaling molecules. Our results provide a scientific basis for further exploration of the molecular mechanism of immune dysfunction caused by acute BVDV infection.

## Materials and Methods

### Ethics Statement

This study was carried out in accordance with the principles of the Basel Declaration and recommendations followed by the guidelines set from College of Animal Science and Veterinary Medicine, HeiLongJiang Bayi Agricultural University. The protocol was approved by the Management Committee of the Experimental Animal Center of Heilongjiang Bayi Agricultural University.

### Animals

Five healthy 1-year-old calvesfrom a Holstein dairy farm located in the city of Daqing in Heilongjiang Province, China were used for blood collection. The cattle were confirmed to be negative for BVDV antibody and BVDV as measured by antibody and antigen-capture ELISA test kits (IDEXX Laboratories, Westbrook, ME, USA) and negative for BLV, IBRV, and BIV infection as measured by PCR as previously described (15–18).

### PBMC Preparation, BVDV Infection, and PBL Subsets Isolation

PBMC were isolated from fresh-heparinized venous blood of cattle by standard Ficoll/Hypaque density gradient centrifugation (Sigma, St. Louis, MO, USA). PBMC (1 × 10^7^/well) were plated on flat-bottom 6-well culture plates in RPMI-1640 (Gibco, Carlsbad, CA, USA) supplemented with 100 units/mL penicillin, 100 μg/mL streptomycin, 1% Glutamax-1 (Invitrogen, Carlsbad, CA, USA) and 10% fetal bovine serum (FBS) (Gibco, Carlsbad, CA, USA) free of BVD virus and antibodies. Cells were infected with the CP or NCP BVDV at a multiplicity of infection (MOI) of 0.01 (with viral copies of 1 × 10^3^ and cell numbers of 1 × 10^5^). The CP BVDV-1a (strain NADL, No. VR-534) was from the American Type Culture Collection (ATCC). The NCP BVDV-1b (strain KD) was isolated from the BVDV persistently infected (PI) cattle in Daqing of China, and identified and preserved by Heilongjiang Provincial Engineering Technology Research Center for Prevention and Control of Cattle Diseases. The infected and uninfected PBMC were incubated at 37°C for 96 h with 5% CO_2_ in the presence of 10 ng/mL phorbol 12-myristate acetate (PMA) and 500 ng/mL ionomycin (Sigma, St. Louis, MO, USA). The PBMC were then individually incubated with monoclonal antibodies against either bovine CD3 (ab16669, Abcam, Cambridge, UK), CD4 (MCA834GA, Bio-Rad, CA, USA), CD8 (MCA837GA, Bio-Rad, CA, USA), CD21 (MCA1424GA, Bio-Rad, CA, USA) or CD14 (MCA2678GA, Bio-Rad, CA, USA) for 30 min at 4°C followed by the addition of magnetic beads conjugated with rabbit anti-IgG, mouse anti-IgG1 or IgG2a+b (Miltenyi Biotech, Auburn, CA). CD3^+^CD4^+^ T cells, CD8^+^ T cells, CD21^+^ B cells and CD14^+^ monocytes were positively selected using a magnetic cell separation technique according to the manufacturer's instructions. Highly purified PBL subsets (> 90%) were used for the analysis of the PD-1 expression.

### Analysis of PD-1 Expression on PBL Subsets

The protein expressions of PD-1 on PBL subsets were measured by western blot analysis at 96 h post-infection (hpi) (14). Total protein was extracted, respectively from CD4^+^ T cells, CD8^+^ T cells, and CD21^+^ B cells with 100–150 μl RIPA buffer (Beytime, HangZhou, China) containing 15 mM PMSF (Beytime, HangZhou, China). Protein concentration was determined using the BCA Protein Assay Kit (Beytime, HangZhou, China) according to the manufacturer's instructions. Approximately 30 μg of total protein was separated by SDS-PAGE and transferred onto a PVDF membrane (0.45 μm, Millipore, Germany). Membranes were blocked with 5% non-fat milk in TBST (Tris-HCl, NaCl, and Tween 20) for 1–2 h at room temperature and then incubated overnight at 4°C with monoclonal antibody (mAb) against PD-1 (ab52587, 1:500, Abcam) or β-actin (60008-1-lg, 1:15000, Proteintech) which was used as an internal control. Membranes were rinsed with TBST three times for 15 min each and incubated with horseradish peroxidase (HRP)-conjugated affinipure goat anti-mouse IgG (H+L) (SA00001-1, 1:8000, Proteintech) for 1 h at room temperature. The membranes were then rinsed as above and treated with chemiluminescent HRP substrate (P90719, Millipore, Germany) that was detected with a chemiluminescence detector (Bio-Rad, CA, USA). Finally, the expression of each protein was analyzed with GraphPad Prism version 6.0 (GraphPad Software).

### PD-1 Blockade Assay

To assess the effect of PD-1 pathway on apoptosis, viral replication and signaling molecules of BVDV-infected CD4^+^ and CD8^+^ T cells, a blocking assay was performed using anti-PD-1 mAb as described in the previous report (11, 16). PBMC were infected with CP or NCP BVDV, respectively, and then cultured at 37°C for 96 h in media supplemented with 10 ng/mL PMA and 500 ng/mL ionomycin in the presence of 10 μg/mL anti-PD-1 antibody (Abcam, Cambridge, UK) for detection of apoptosis, viral replication and signaling molecules. Anti-PD-1 antibody was added only once at the beginning.

### Cell Apoptosis Measurement Using Flow Cytometry

The CD4^+^ and CD8^+^ T cells were selected, respectively, from the PBMC using magnetic cell separation technique. These cells were stained using Annexin-V-FITC apoptosis Kit with propidium iodide for 15 min at room temperature in the dark according to the manufacturer's instructions (Beyotime, Shanghae, China) and analyzed immediately on a CytoFLEX flow cytometer (Beckman Coulter, USA).

### Cell Proliferation Assay

The CD4^+^ and CD8^+^ T cells were individually co-cultured with CD14^+^ monocytes and infected with CP or NCP BVDV, respectively. The cells were cultured at 37°C in media containing 10 ng/mL PMA and 500 ng/mL ionomycin in the presence of 10 μg/mL anti-PD-1 antibody for 7 days in an incubator supplemented with 5% CO_2_. Anti-PD-1 antibody was added only once at the beginning. The infected cells without antibody were used as the controls. Cell proliferation was detected using cell counting kit-8 (Dojindo Laboratories, Kumamoto, Japan) according to the manufacturer's instructions. The density of cells in each well was measured according to the absorbance at 450 nm every 24 h.

### Effect of PD-1 Blockade on Virus Replication

To evaluate the effects of PD-1 blockade on the time course of virus replication in CD4^+^ and CD8^+^ T cells, nucleotide sequences of 5′ non-coding region (NCR) of BVDV was amplified and cloned into a pMD18-T vector for the establishment of qRT-PCR as standard DNA (11). Primers were used as follow, 5′-GAG TAC AGG GTA GTC GTC AG-3′ and 5′-CTC TGC AGC ACC CTA TCA GG-3′ for 5′NCR, and 5′-CGC ACC ACT GGC ATT GTC AT-3′ and 5′-TCC AAG GCG ACG TAG CAG AG-3′ for β-actin. The recombinant plasmid pMD18-T/5′NCR was serially diluted 10-fold in TE buffer, pH 8.0, from 10^−1^ to 10^−7^. Each dilution of them was tested in triplicate and used as an amplification template to construct standard curves to determine the copy number of the BVDV 5′NCR gene. Quantitative real-time PCR was performed in the CFX96 Touch Real-Time PCR Detection System (Bio-Rad, Hercules, CA, USA) using SYBR Premix Ex Taq II (TaKaRa Biotechnology, Dalian, China) following the manufacturer's instructions. Each cDNA sample from PBMCs suspension was tested in triplicate; the cycling conditions consist of initial template denaturing at 95°C for 30 s, followed by amplification of template for 45 cycles of 95°C for 5 s, 60°C for 30 s and 72°C for 30 s. A final melting curve analysis was performed from 65 to 95°C at a rate of 0.1°C/s (continuous acquisition), with a final cooling to 40°C over 10 s. The results are shown as the mean copy number per milliliter of cell suspension.

Meanwhile, to determine if most cells were infected by the virus, virus-infected CD4^+^ and CD8^+^ T cells were analyzed by confocal laser scanning microscopy (CLSM) at 96 hpi. The cells were collected by centrifugation at 1,000 g for 5 min at 4°C, washed once with PBS, and resuspended with 20 μL PBS. The suspension was dripped on poly-L-lysine-coated coverslip and dried at 37°C for 15 min. Then, the cells were fixed with cold acetone, permeabilized with 0.3% Triton X-100, blocked with 5% non-fat milk, and incubated with a mouse anti-BVDV Npro polyclonal antibody (1:100) and an ATP1A1 antibody (14418-1-AP, 1:300; Proteintech) overnight at 4°C. ATP1A1 antibody was used to stain the cell membranes. The cells were then incubated with an Alexa fluor 488-conjugated affinipure donkey anti-rabbit IgG (H+L) (SA00006-6, 1:200; Proteintech) and an Alexa fluor 594-conjugated affinipure donkey anti-mouse IgG (H+L) (SA00006-7, 1:200; Proteintech). The nuclei were stained with 4′,6-diamidino-2-phenylindole (DAPI), and the coverslips were observed using a laser scanning confocal microscope (TCS SP2; Leica).

### Western Blot Analysis of PD-1 Downstream Signaling Molecules

PD-1 downstream signaling molecules in CD4^+^ and CD8^+^ T cells were measured by Western blot analysis at 96 hpi. The primary antibodies were PI3K (ab227204, 1:1000, Abcam), p-PI3K (ab182651, 1:1000, Abcam), AKT (#9272, 1:1000, Cell Signaling Technology, USA), p-AKT (Ser473) (#9271, 1:1000, Cell Signaling Technology), caspase 9 (ab202068, 1:1000, Abcam), caspase 3 (19677-1-AP, 1:500, Proteintech), ERK (#4695S, 1:1000, Cell Signaling Technology), p-ERK (Thr202/Tyr204) (#9101, 1:1000, Cell Signaling Technology), mTOR (ab2732, 1:1000, Abcam), p-mTOR (ab84400, 1:1000, Abcam), and β-actin (60008-1-lg, 1:15000, Proteintech), which was used as an internal control. The secondary antibodies were HRP-conjugated affinipure goat anti-mouse IgG (H+L) (SA00001-1, 1:8000, Proteintech) and HRP-conjugated affinipure goat anti-rabbit IgG (H+L) (SA00001-2, 1:8000, Proteintech).

### Statistical Analysis

All data were expressed as mean ± SD and analyzed using student's unpaired *t*-test, one-way ANOVA and two-way ANOVA using GraphPad Prism version 6.0 (GraphPad Software). A *p* < 0.05 indicated a statistically significant difference. All samples were assayed in triplicate.

## Results

### Expression of PD-1 on PBL Subsets After BVDV Infection

**Figure 1:**
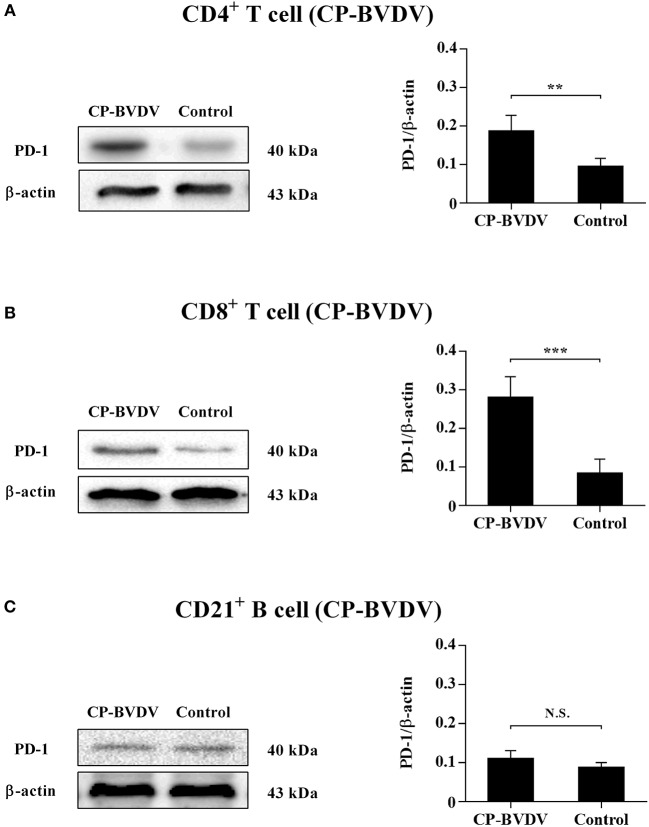
Western blot analysis of PD-1 protein expression after CP BVDV infection. Shown are the representative western blot results and densitometric analyses of five independent experiments showing the expression of PD-1 protein on CD4^+^ T cells **(A)**, CD8^+^ T cells **(B)**, and CD21^+^ B cells **(C)**. The uninfected CD4^+^ T cells, CD8^+^ T cells, and CD21^+^ B cells were used as the controls. N.S., not significant, ***p < 0.001, **p < 0.01. Data are presented as mean ± SD (*n* = 5 per group).

**Figure 2:**
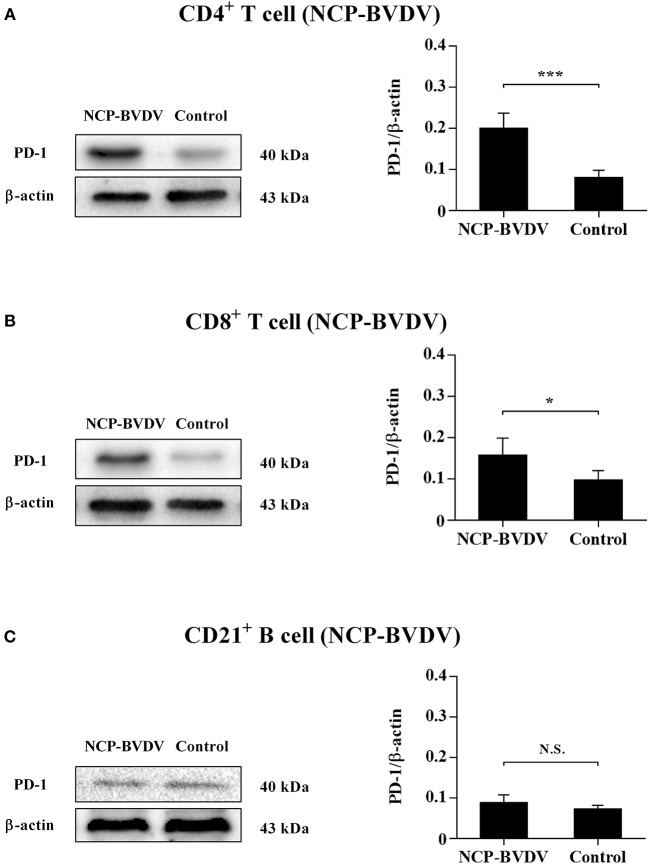
Western blot analysis of PD-1 protein expression after NCP BVDV infection. Shown are the representative western blot results and densitometric analyses of five independent experiments showing the expression of the PD-1 protein on CD4^+^ T cells **(A)**, CD8^+^ T cells **(B)**, and CD21^+^ B cells **(C)**. The uninfected CD4^+^ T cells, CD8^+^ T cells, and CD21^+^ B cells were used as the controls. N.S., not significant, ***p < 0.001, *p < 0.05. Data are presented as mean ± SD (*n* = 5 per group).

Our previous report (14) has confirmed that PD-1 expression was significantly upregulated on both the CP BVDV and the NCP BVDV infected PBL compared with uninfected PBL. To study the protein expressions of PD-1 on PBL subsets after infection with BVDV, western blot analysis was performed in this study. The results showed that there was a significant increase in PD-1 expression on CD4^+^ (CP BVDV, *p* < 0.01, Figure 1A; NCP BVDV, *p* < 0.001, Figure 2A) and CD8^+^ T cells (CP BVDV, *p* < 0.001, Figure 1B; NCP BVDV, *p* < 0.05, Figure 2B) after both the CP BVDV and the NCP BVDV infection. However, there was no significant increase in PD-1 expression on CD21^+^ B cells (Figures 1C, 2C). Original data is shown in Figures S20–S22 in Supplementary Material.

### Detection of Apoptosis in CD4^+^ and CD8^+^ T Cells

**Figure 3:**
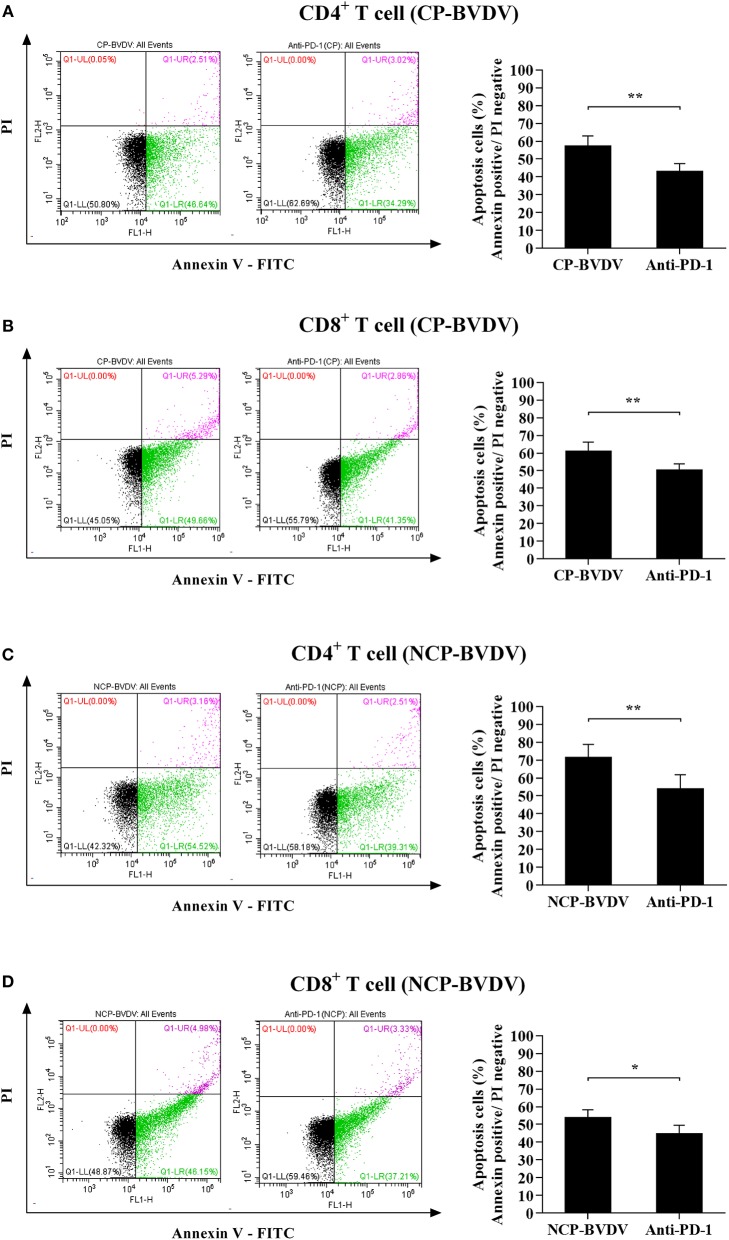
Effect of the PD-1 blockade on the apoptosis of BVDV-infected CD4^+^ and CD8^+^ T cells. Shown are the results of flow cytometry analyses of apoptosis of CP BVDV-infected CD4^+^ T cells **(A)** and CD8^+^ T cells **(B)**, as well as NCP BVDV-infected CD4^+^ T cells **(C)**, and CD8^+^ T cells **(D)**. The infected CD4^+^ and CD8^+^ T cells without antibody were used as the controls. **p < 0.01, *p < 0.05. Data are presented as mean ± SD (*n* = 5 per group).

To assess the effect of PD-1 pathway on the apoptosis of BVDV-infected CD4^+^ and CD8^+^ T cells, we investigated the cell apoptosis by flow cytometric analysis. The results showed that the proportion of apoptotic cells was significantly reduced in the infected CD4^+^ (CP BVDV, *p* < 0.05, Figure 3A; NCP BVDV, *p* < 0.05, Figure 3C) and CD8^+^ T cells (CP BVDV, *p* < 0.01, Figure 3B; NCP BVDV, *p* < 0.05, Figure 3D) by PD-1 blockade.

### Measurement of CD4^+^ and CD8^+^ T Cells Proliferation

**Figure 4:**
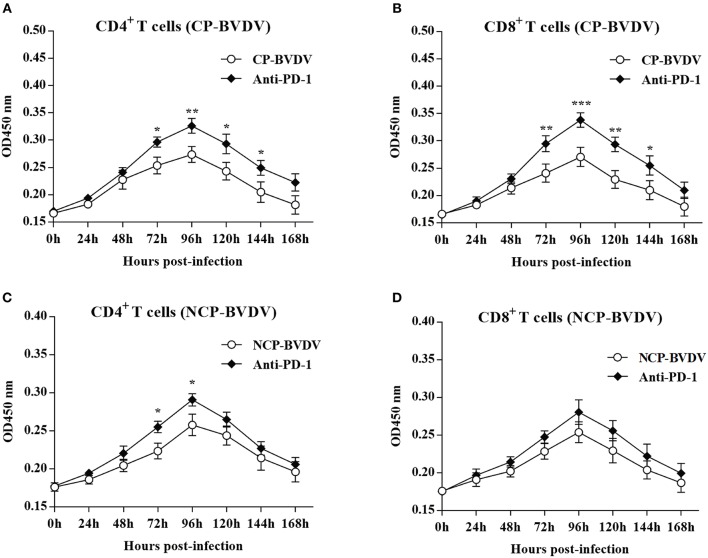
Effect of PD-1 blockade on the proliferation of BVDV-infected CD4^+^ and CD8^+^ T cells. Shown are the results of proliferation assay of CP BVDV-infected CD4^+^ T cells **(A)** and CD8^+^ T cells **(B)** as well as NCP BVDV-infected CD4^+^ T cells **(C)** and CD8^+^ T cells **(D)**. The infected CD4^+^ and CD8^+^ T cells without antibody were used as the controls. ***p < 0.001, **p < 0.01, *p < 0.05. Data are presented as mean ± SD (*n* = 5 per group).

To investigate the effect of PD-1 pathway on the proliferation of BVDV-infected CD4^+^ and CD8^+^ T cells, we detected the cell proliferation using cell counting kit-8. PD-1 blockade significantly restored proliferation of the CP BVDV infected CD4^+^ and CD8^+^ T cells from 72 to 144 hpi (Figures 4A,B) and the proliferation of the NCP BVDV infected CD4^+^ T cells at 72 and 96 hpi (*p* < 0.05, Figure 4C), but not NCP BVDV infected CD8^+^ T cells (Figure 4D).

### Viral Replication

**Figure 5:**
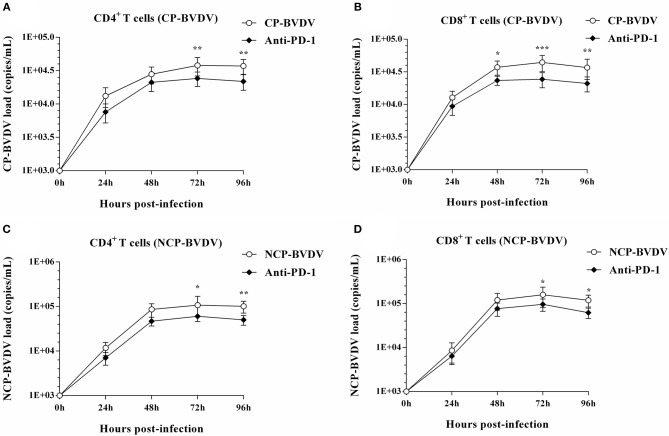
Effect of PD-1 blockade on viral replication in CD4^+^ and CD8^+^ T cells. The time course of virus replication in CP BVDV-infected CD4^+^ T cells **(A)**. The time course of virus replication in CP BVDV-infected CD8^+^ T cells **(B)**. The time course of virus replication in NCP BVDV-infected CD4^+^ T cells **(C)**. The time course of virus replication in NCP BVDV-infected CD8^+^ T cells **(D)**. The BVDV-infected cells without antibody were used as control groups. *p < 0.05, **p < 0.01, ***p < 0.001 vs. control group. Data are presented as mean ± SD (*n* = 5 per group).

**Figure 6:**
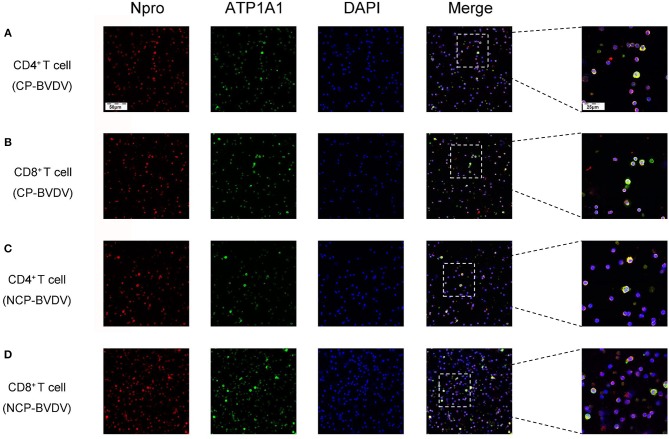
CLSM analysis of virus-infected CD4^+^ and CD8^+^ T cells at 96 hpi. CP BVDV-infected CD4^+^ T cells **(A)**. CP BVDV-infected CD8^+^ T cells **(B)**. NCP BVDV-infected CD4^+^ T cells **(C)**. NCP BVDV-infected CD8^+^ T cells **(D)**. The red color denotes BVDV Npro, the green color denotes ATP1A1 and the blue color denotes DAPI.

To assess the effects of PD-1 blockade on the time course of virus replication, we investigated the BVDV load in the infected CD4^+^ and CD8^+^ T cells by quantitative real-time PCR from 24 to 96 hpi. After treatment with an anti-PD-1 antibody, the replication of the CP BVDV and NCP BVDV were significantly inhibited at 72 and 96 hpi in infected CD4^+^ (CP BVDV, Figure 5A; NCP BVDV, Figure 5C) and CD8^+^ T cells (CP BVDV, Figure 5B; NCP BVDV, Figure 5D). Moreover, observation using CLSM (confocal laser scanning microscopy) intuitively showed that most CD4^+^ and CD8^+^ T cells were infected by virus at 96 hpi (CP BVDV, Figures 6A,B; NCP BVDV, Figures 6C,D).

### Effects of PD-1 Blockade on the Expression of Downstream Signaling Molecules

**Figure 7:**
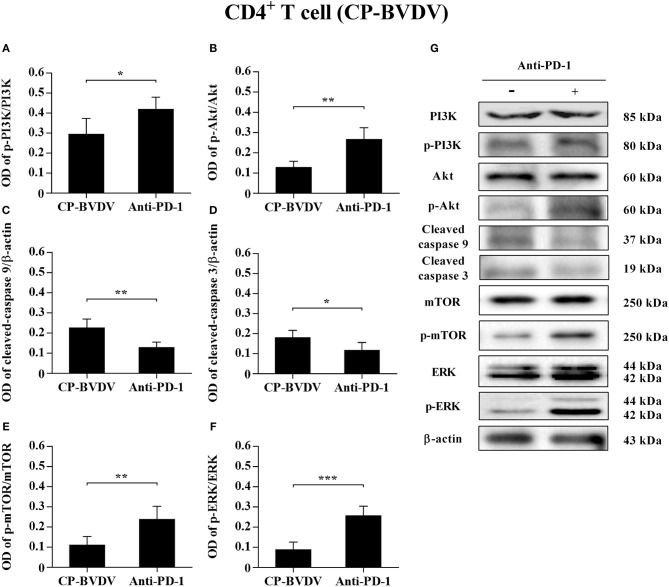
Effect of PD-1 blockade on the PD-1 downstream signaling molecules in CP BVDV-infected CD4^+^ T cells. Shown are the results of densitometric analyses of the levels of p-PI3K **(A)**, p-Akt **(B)**, cleaved-caspase 9 **(C)**, cleaved-caspase 3 **(D)**, p-mTOR **(E)**, p-ERK **(F)** in bar graph format as well as the representative results **(G)** of Western blot analysis of PI3K, p-PI3K, Akt, p-Akt, cleaved-caspase 9, cleaved-caspase 3, mTOR, p-mTOR, ERK, p-ERK, and β-actin. The infected CD4^+^ and CD8^+^ T cells without antibody were used as control groups. ***p < 0.001, **p < 0.01, *p < 0.05. Data are presented as mean ± SD (*n* = 5 per group).

**Figure 8:**
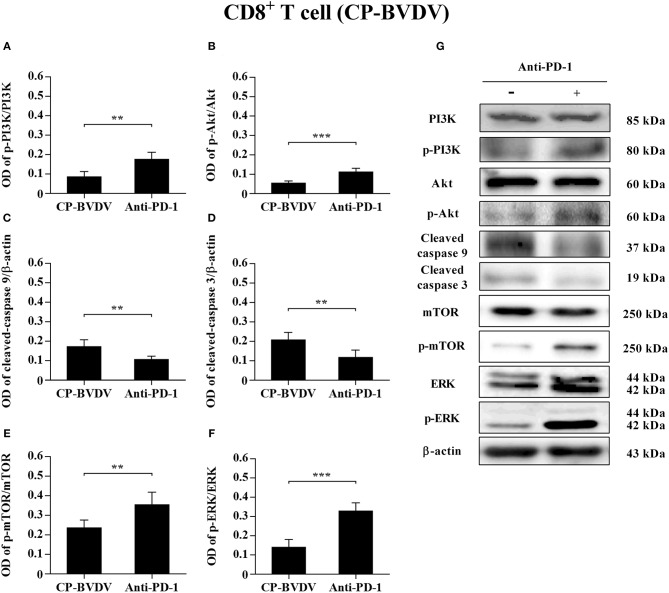
Effect of PD-1 blockade on downstream signaling molecules in CP BVDV-infected CD8^+^ T cells. Shown are the results of densitometric analyses of the levels of p-PI3K **(A)**, p-Akt **(B)**, cleaved-caspase 9 **(C)**, cleaved-caspase 3 **(D)**, p-mTOR **(E)**, p-ERK **(F)** in bar graph format as well as the representative results **(G)** of Western blot analysis of PI3K, p-PI3K, Akt, p-Akt, cleaved-caspase 9, cleaved-caspase 3, mTOR, p-mTOR, ERK, p-ERK, and β-actin. The infected CD4^+^ and CD8^+^ T cells without antibody were used as control groups. ***p < 0.001, **p < 0.01. Data are presented as mean ± SD (*n* = 5 per group).

**Figure 9:**
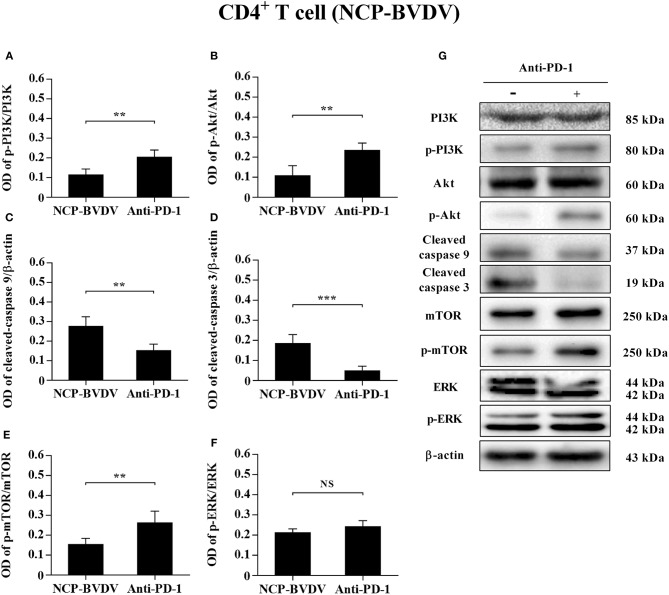
Effect of PD-1 blockade on downstream signaling molecules in NCP BVDV-infected CD4^+^ T cells. Shown are the results of densitometric analyses of the levels of p-PI3K **(A)**, p-Akt **(B)**, cleaved-caspase 9 **(C)**, cleaved-caspase 3 **(D)**, p-mTOR **(E)**, p-ERK **(F)** in bar graph format as well as the representative results **(G)** of Western blot analysis of PI3K, p-PI3K, Akt, p-Akt, cleaved-caspase 9, cleaved-caspase 3, mTOR, p-mTOR, ERK, p-ERK, and β-actin. The infected CD4^+^ and CD8^+^ T cells without antibody were used as control groups. N.S., not significant, ***p < 0.001, **p < 0.01. Data are presented as mean ± SD (*n* = 5 per group).

**Figure 10:**
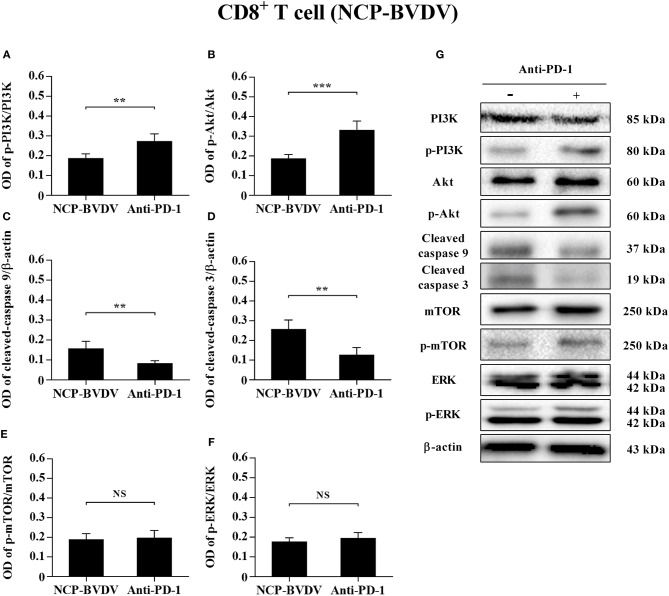
Effect of PD-1 blockade on the PD-1 downstream signaling molecules in NCP BVDV-infected CD8^+^ T cells. Shown are the results of densitometric analyses of the levels of p-PI3K **(A)**, p-Akt **(B)**, cleaved-caspase 9 **(C)**, cleaved-caspase 3 **(D)**, p-mTOR **(E)**, p-ERK **(F)** in bar graph format as well as the representative results **(G)** of Western blot analysis of PI3K, p-PI3K, Akt, p-Akt, cleaved-caspase 9, cleaved-caspase 3, mTOR, p-mTOR, ERK, p-ERK, and β-actin. The infected CD4^+^ and CD8^+^ T cells without antibody were used as control groups. N.S., not significant, ***p < 0.001, **p < 0.01. Data are presented as mean ± SD (*n* = 5 per group).

To determine the effects of PD-1 blockade on the expression and phosphorylation of downstream signaling molecules, western blot analysis was performed. In the CP BVDV infected CD4^+^ and CD8^+^ T cells, the expression levels of p-PI3K, p-Akt, p-mTOR and p-ERK were significantly upregulated by PD-1 blockade (CD4^+^ T cells, Figures 7A,B,E–G; CD8^+^ T cells, Figures 8A,B,E–G). Meanwhile, the expression levels of cleaved-caspase 9 and cleaved-caspase 3 were significantly downregulated (CD4^+^ T cells, Figures 7C,D; CD8^+^ T cells, Figures 8C,D). Furthermore, in the NCP BVDV infected CD4^+^ and CD8^+^ T cells, we observed a significant increase in p-PI3K and p-Akt (CD4^+^ T cells, Figures 9A,B,G; CD8^+^ T cells, Figures 10A,B,G), and a significant decrease in cleaved-caspase 9 and cleaved-caspase 3 by PD-1 blockade (CD4^+^ T cells, Figures 9C,D; CD8^+^ T cells, Figures 10C,D). Remarkably, the expression level of p-mTOR was significantly upregulated in the NCP BVDV infected CD4^+^ T cells (*p* < 0.01, Figure 9E) but not significantly changed in the NCP BVDV infected CD8^+^ T cells (Figure 10E) by PD-1 blockade. In addition, PD-1 blockade had no significant effect on p-ERK expression in the NCP BVDV infected CD4^+^ and CD8^+^ T cells (Figures 9F, 10F). Original data is shown in Figures S1–S19 in Supplementary Material.

## Discussion

Lymphocytes, such as T-helper cells, cytotoxic T cells and B cells, play vital roles in controlling virus infections and eliminating the viruses from the infected cells (19, 20). Regulation of T cell activation requires not only the recognition of antigen by the antigen specific TCR but also co-signaling molecules categorized as co-stimulatory and co-inhibitory molecules (21). PD-1, as a co-inhibitory molecule, inhibits TCR-mediated activation and proliferation, and induces lymphocyte apoptosis and immune dysfunction by binding to its ligand such as PD-L1 (22). In a previous study we reported that PD-1 plays a vital role in peripheral blood lymphopenia and apoptosis caused by acute BVDV infection (14). However, it is unclear what the immunomodulatory effect of the PD-1 pathway is on major PBL subsets. Moreover, the molecular mechanism underlying this effect needs to be further studied. It has been shown that PD-1 was upregulated on CD8^+^ and CD4^+^ T cells and that its expression increased apoptosis of HIV-specific CD8^+^ T cells (23) and showed an inverse correlation with CD4^+^ T cell count (9). Studies on HBV infection demonstrated that blocking PD-1-mediated pathway *in vitro* reduced HBV-specific CD8^+^ T cell apoptosis and IL-10 production (24). In this study, we demonstrated that an increase in PD-1 expression in CD4^+^ and in CD8^+^ T subgroup but not in CD21^+^ B cells when the cells were infected with BVDV. Meanwhile, PD-1 blockade significantly reduced apoptosis and viral load in CD4^+^ and CD8^+^ T cells after BVDV infection. Our results suggest that the PD-1 pathway regulates apoptosis of CD4^+^ and CD8^+^ T cells during the CP BVDV and the NCP BVDV infection *in vitro*.

PD-1 also inhibits BCR-mediated B cell activation and proliferation (25), which leads to the prevention of autoimmune diseases. However, very few studies have been done on PD-1 regulating B cell survival and functions during viral infection. It has been shown that PD-1 expression was increased on CD21^+^ CD27^+^ resting memory B cells in HIV-1-infected patients, which might contribute to a decreased ratio of the resting memory B cells during HIV-1 infection (26). Notably, B cells are also important target of BVDV, and their apoptotic effect on B cells depends on the strain (27). We focused on lymphocyte subsets with high expression of PD-1 in this study. Infection of both CP BVDV and NCP BVDV increased PD-1 expression in CD21^+^ B cells, but these increases were not significant. Infection of both CP BVDV and NCP BVDV may affect PD-1 expression in CD21^+^ subpopulations such as CD21^+^ CD27^+^ resting memory B cells. The relationship between B cell apoptosis and PD-1 need to be further explored.

In fact, the cytoplasmic tail of PD-1 contains an immunoreceptor tyrosine-based switch motif (ITSM) encompassing Y248, which interacts with SH_2_ domain-containing protein-tyrosine phosphatase (SHP-2) and mediates PD-1 inhibitory signal (28). SHP-2 inhibits Lck-mediated phosphorylation of ZAP-70 and initiation of downstream signal pathways such as PI3K/Akt and ERK pathways (29). The PI3K/Akt/mTOR pathway can regulate cell proliferation and apoptosis. Abnormally elevated mTOR expression promotes tumor proliferation and metastasis in many human malignancies (30). The mTOR pathway also plays an important role in innate and acquired immunity. Activated PI3K/Akt/mTOR pathway can improve T lymphocyte metabolism, nutrient uptake and energy production, regulate cell cycle and apoptosis, and affect T lymphocyte activation and immune function (31–34). Blocking the interaction between PD-1 and PD-L1 can reactivate PI3K/Akt/mTOR pathway and restore immune function of exhausted CD8^+^ T cells (35). In addition, the activation of the Ras/MEK/ERK pathway can promote cell protein synthesis and proliferation. PD-1 blockade restored T lymphocyte proliferation and improve its antiviral function by reactivating the Ras/MEK/ERK pathway (29). Caspase 9 is a key pro-apoptotic signaling molecule downstream of the PI3K/Akt pathway and an essential initiator of the mitochondrial apoptosis pathway (36). The PI3K/Akt pathway plays an anti-apoptotic role through inhibiting the caspase 9 signaling cascade (37). In BVDV infected cattle, the expression of caspase 9 and caspase 3 is involved in lymphoid tissues lesions, but whether it directly induces apoptosis in infected cells or whether it is related to the PD-1 pathway remains unclear (38). In our study, by measuring the levels of p-PI3K, p-Akt, p-mTOR, p-ERK, cleaved-caspase 9, and cleaved-caspase 3, we confirmed that CP BVDV infection can inhibit the proliferation of CD4^+^ and CD8^+^ T cells and induce apoptosis via PD-1-mediated repression of PI3K/Akt/mTOR and ERK pathways and activation of the caspase 9/caspase 3 pathway. NCP BVDV infection inhibits the proliferation of CD4^+^ T cells by suppressing of PI3K/Akt/mTOR pathway, and induces the apoptosis of CD4^+^ and CD8^+^ T cells by activating the caspase 9/caspase 3 pathway.

Overexpression of PD-1 can inhibit proliferation of virus-specific CD4^+^ and CD8^+^ T cells (9, 39), and PD-1 blockade can restore proliferation and anti-viral immune functions of T cells (24, 40). However, PD-1 blockade in NCP BVDV infected cells did not significantly affect proliferation and p-mTOR and p-ERK levels of CD8^+^ T cells. Meanwhile, PD-1 blockade had a more significant effect on the decrease of viral load in CP BVDV infected CD8^+^ T cells than in NCP BVDV infected CD8^+^ T cells. It is known that activation and proliferation of T cells are negatively regulated by many co-inhibitory molecules, such as Tim-3 (41) and CTLA-4 (42). Tim-3 expression was upregulated on HIV-1 infected CD8^+^ T cells and associated with the inhibition of T cell proliferation and the impairment of ERK signaling (43). Blocking the co-inhibitory molecule Tim-3 with antibodies can enhance the proliferation of HIV-specific CD8^+^ T cells (44). CTLA-4 inhibits T cell proliferation and IL-2 production by suppressing TCR/CD28-induced ERK and JNK activation (45). In addition, the combined blockade of PD-1/CTLA-4 can synergistically reverse exhaustion of HCV-specific CD8^+^ T cells (46). Notably, both HCV and BVDV belong to the *Flaviviridae* family. In addition, previous study on PD-1 blockade has confirmed the synergistic effect of PD-1 and other co-inhibitory molecules on T cells dysfunction during HIV (44) and HCV (45) infections. However, it is unclear whether PD-1 cooperates with other co-inhibitory molecules to regulate T cells dysfunction during BVDV infection, especially during NCP BVDV infection. Consistently, the blocking assays in this study also revealed that PD-1 is associated with T cells dysfunction, but the possible role of other co-inhibitory molecules needs to better evaluate by more optimized experimental designs, such as the combination of blocking assay and silencing assay.

In summary, we have demonstrated enhancement of PD-1 expression on BVDV-infected CD4^+^ and CD8^+^ T cells, but not B cells, following infection with CP BVDV and NCP BVDV *in vitro*. PD-1 blockade significantly reduced apoptosis of T cells and increased proliferation of CD4^+^ and CD8^+^ T cells after CP BVDV infection and increased proliferation of CD4^+^ T cells after NCP BVDV infection. We also reported the involvement of PI3K/Akt/mTOR, ERK, and caspase 9/caspase 3 pathways in CP BVDV induction of apoptosis and inhibition of CD4^+^ and CD8^+^ T cell proliferation. These pathways were shown to have a similar effect on CD4^+^ T cells following NCP BVDV infection. Remarkably, ERK is involved in the regulation mechanism PD-1 mediated only when the cells are infected with CP BVDV. In addition, a weakness of this paper is that bovine WBC were not taken during the experiment. The situation *in vivo* remains to be further studied and confirmed.

## Data Availability Statement

The datasets generated for this study are available on request to the corresponding author.

## Ethics Statement

The protocol was approved by the Management Committee of the Experimental Animal Center of Heilongjiang Bayi Agricultural University.

## Author Contributions

YL: data collection, data analysis and interpretation, and drafting the article. WH and CW: data collection and data analysis and interpretation. SLia and LW: data analysis and interpretation and drafting the article. YL and BX: critical revision of the article. SLiu and NC: data analysis and interpretation and critical revision of the article. SY: conception or design of the work and critical revision of the article. ZZ: conception or design of the work, data analysis and interpretation, drafting the article, and critical revision of the article.

### Conflict of Interest

The authors declare that the research was conducted in the absence of any commercial or financial relationships that could be construed as a potential conflict of interest.
