# Supplementary figures and images for: PD-1-Mediated PI3K/Akt/mTOR, Caspase 9/Caspase 3 and ERK Pathways Are Involved in Regulating the Apoptosis and Proliferation of CD4+ and CD8+ T Cells During BVDV Infection in vitro
Source: Front Immunol. 2020 Mar 17;11:467. doi: 10.3389/fimmu.2020.00467 (PMC7089960; doi:10.3389/fimmu.2020.00467)

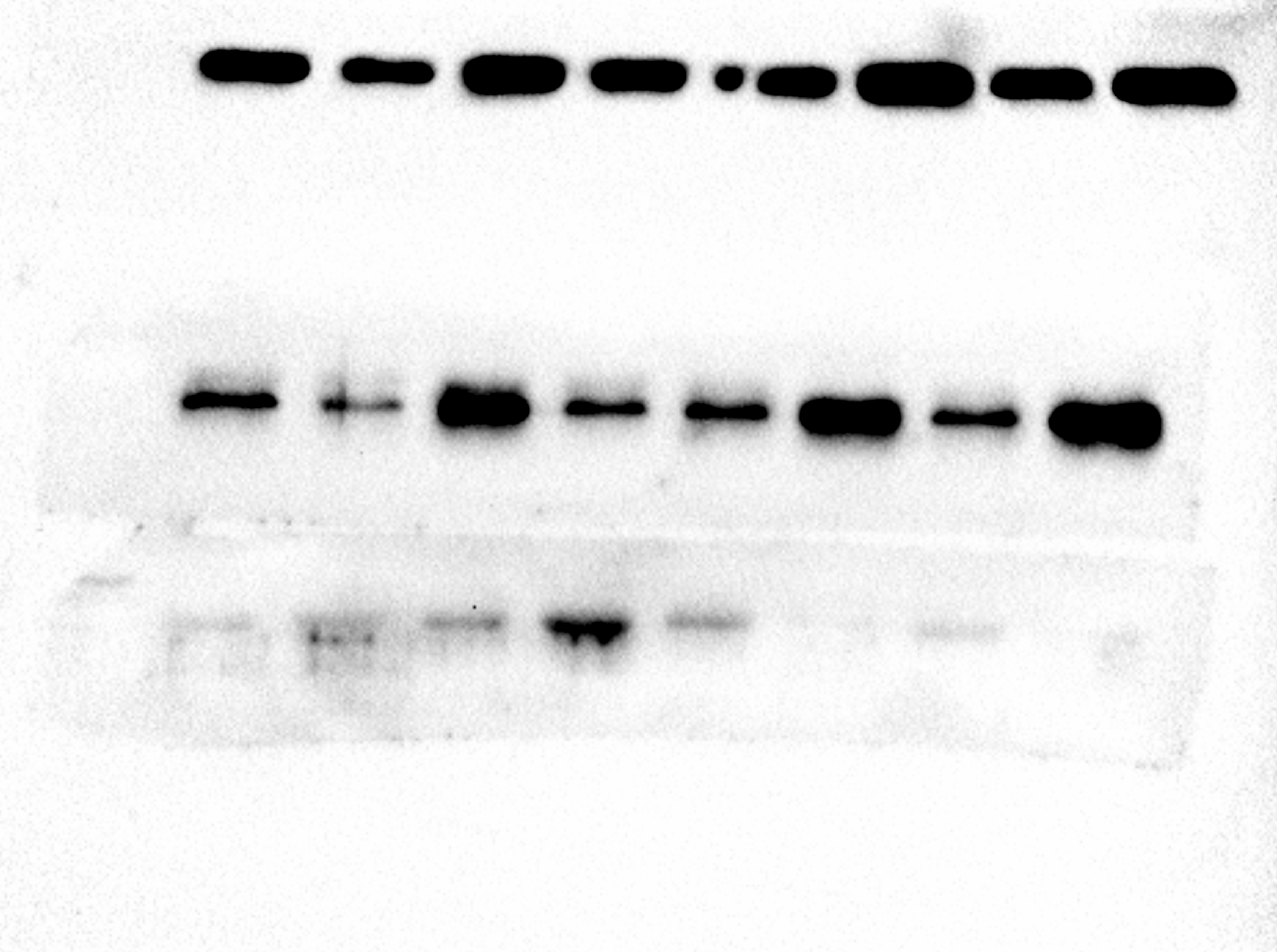

Supplement: Supplementary file 1 [file Data_Sheet_1.zip › Supplementary Data Sheets 4/Figure S1.tif]

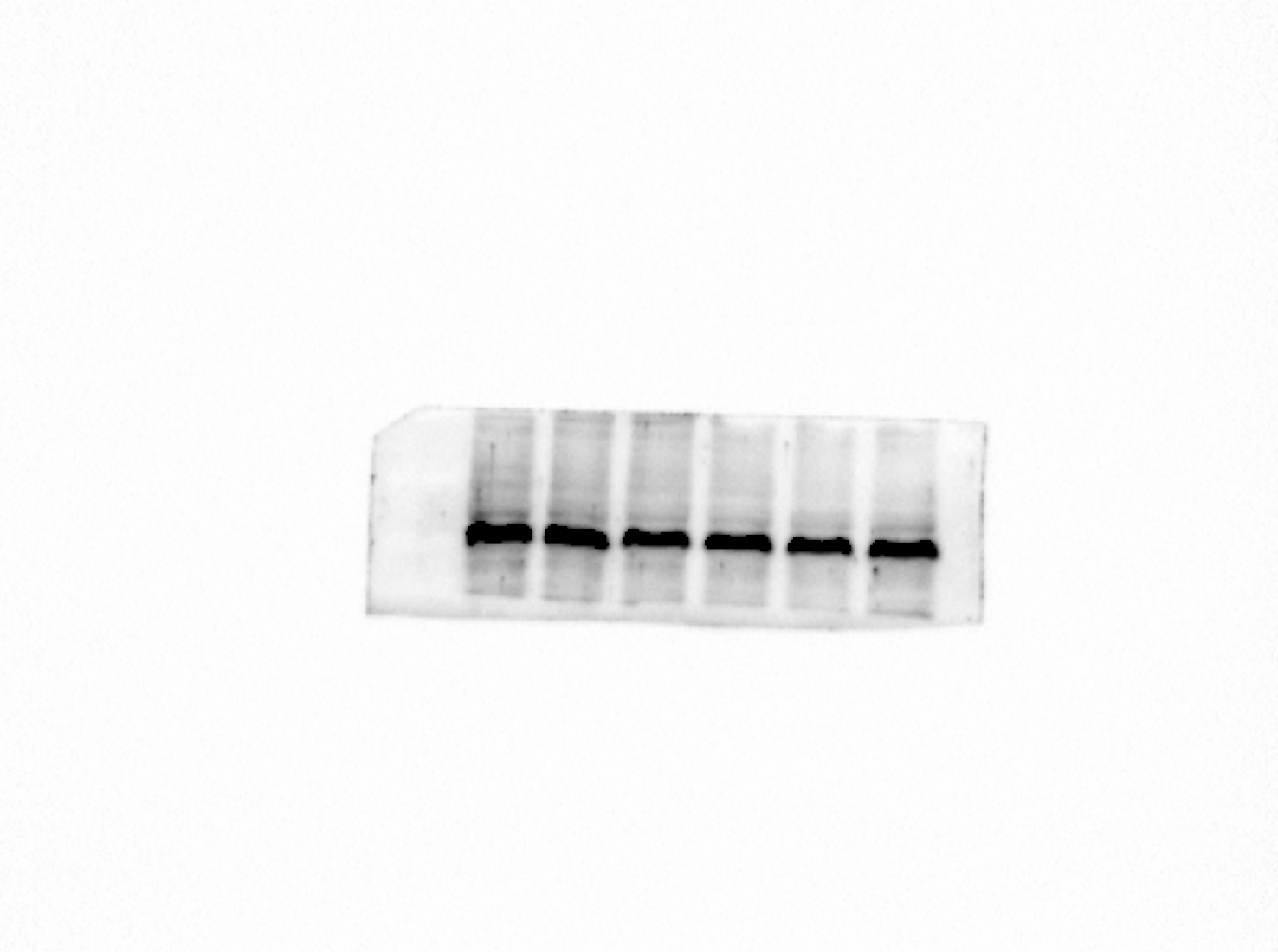

Supplement: Supplementary file 1 [file Data_Sheet_1.zip › Supplementary Data Sheets 4/Figure S10.tif]

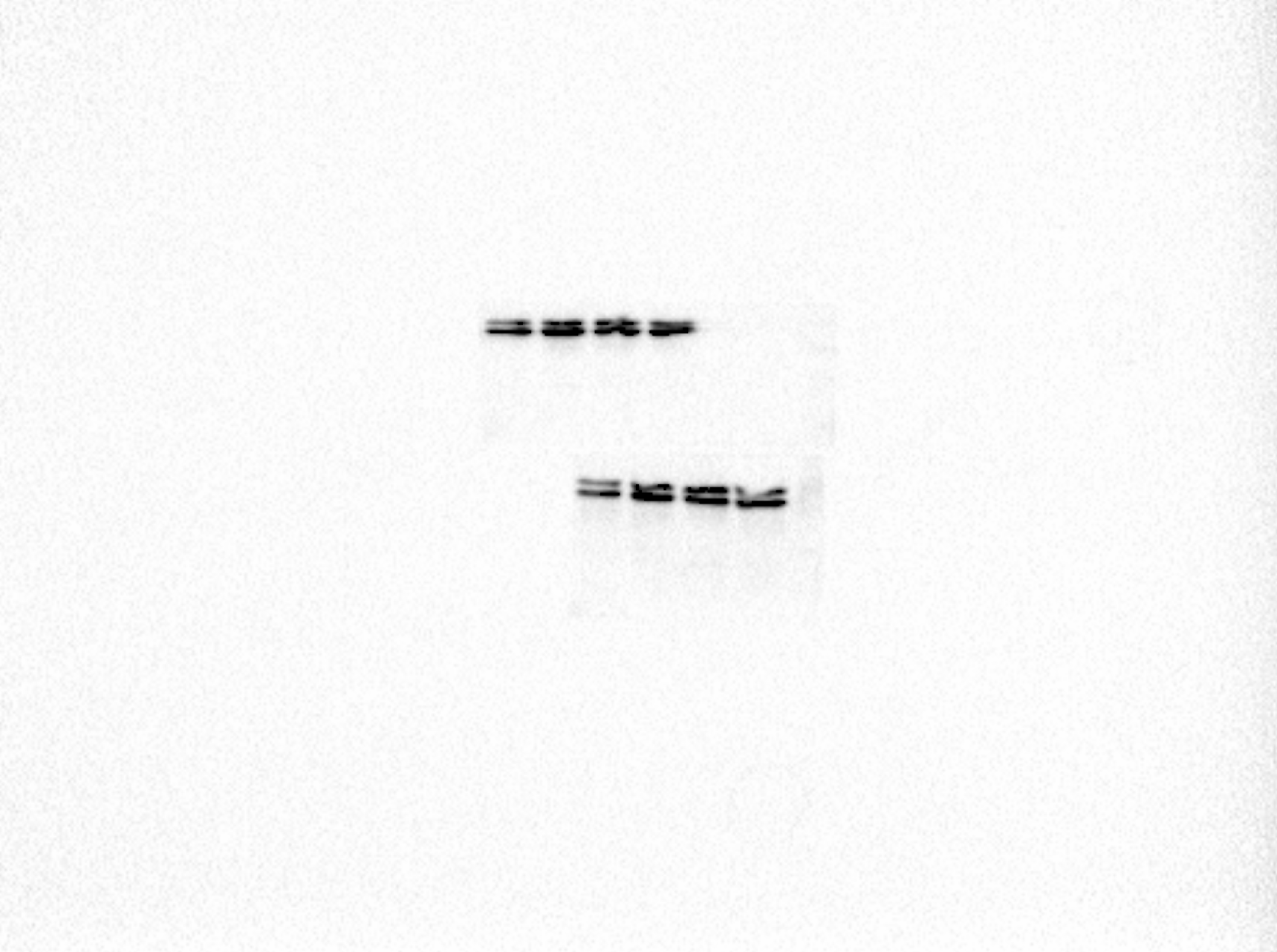

Supplement: Supplementary file 1 [file Data_Sheet_1.zip › Supplementary Data Sheets 4/Figure S2.tif]

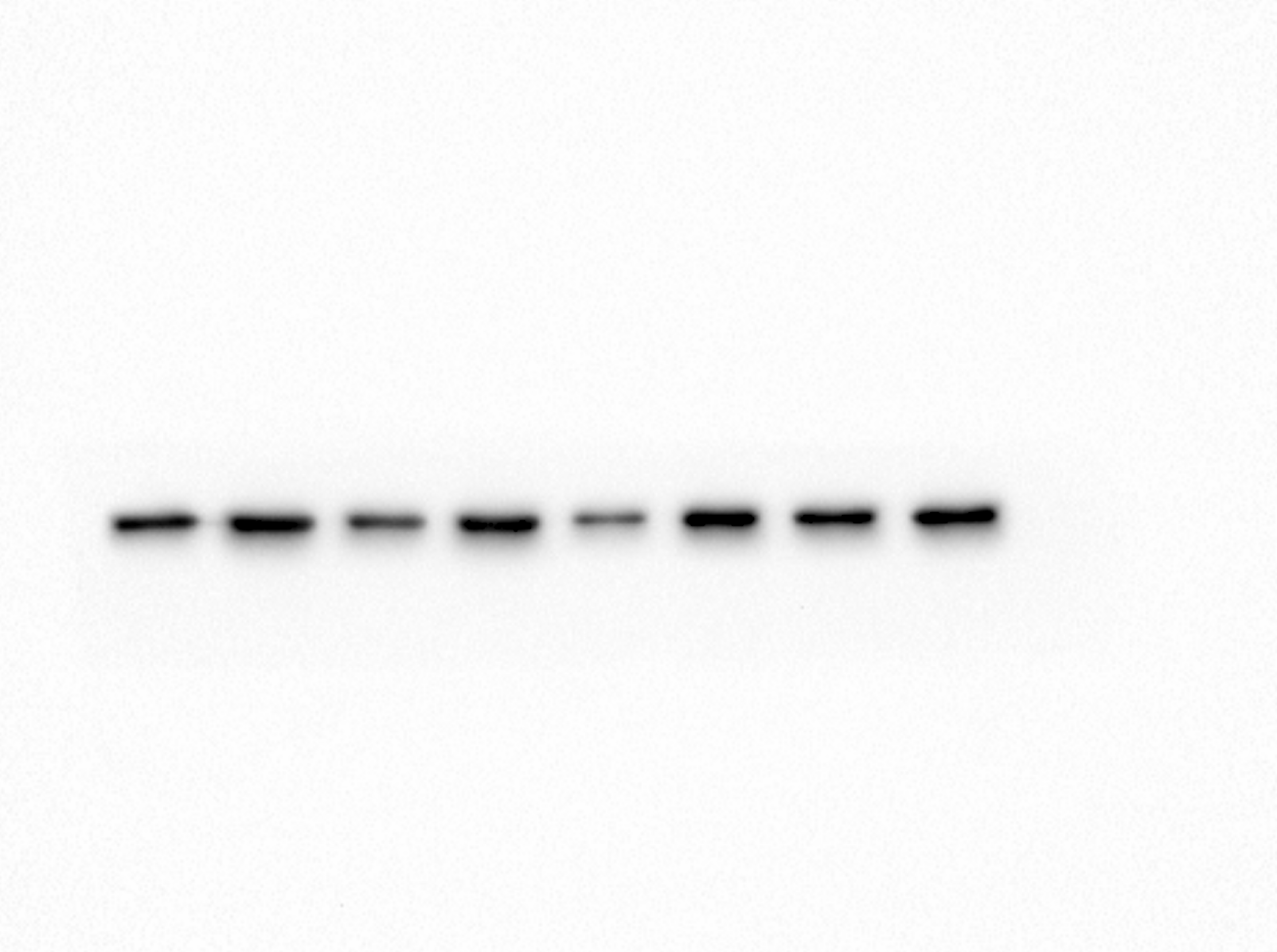

Supplement: Supplementary file 1 [file Data_Sheet_1.zip › Supplementary Data Sheets 4/Figure S3.tif]

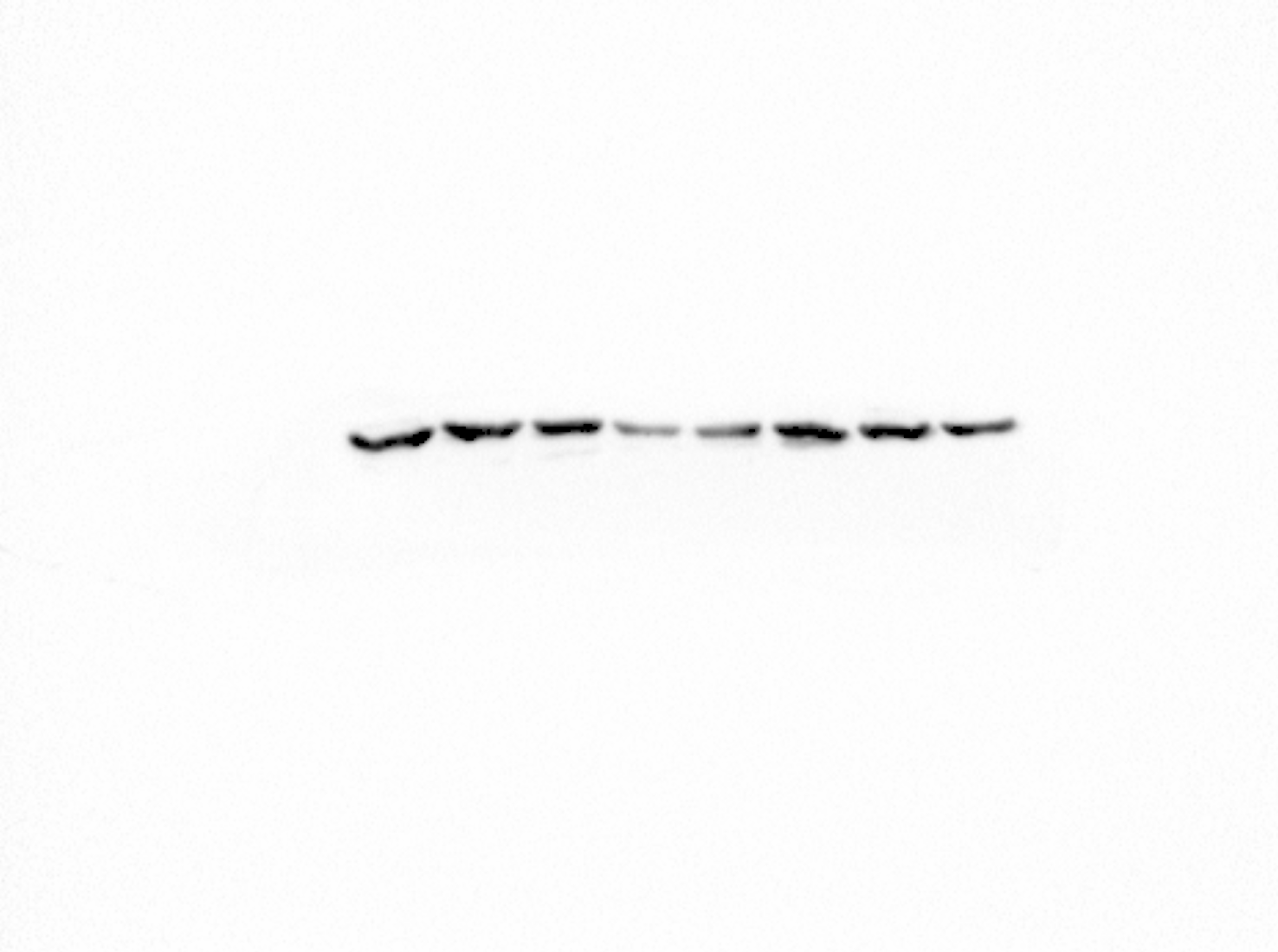

Supplement: Supplementary file 1 [file Data_Sheet_1.zip › Supplementary Data Sheets 4/Figure S4.tif]

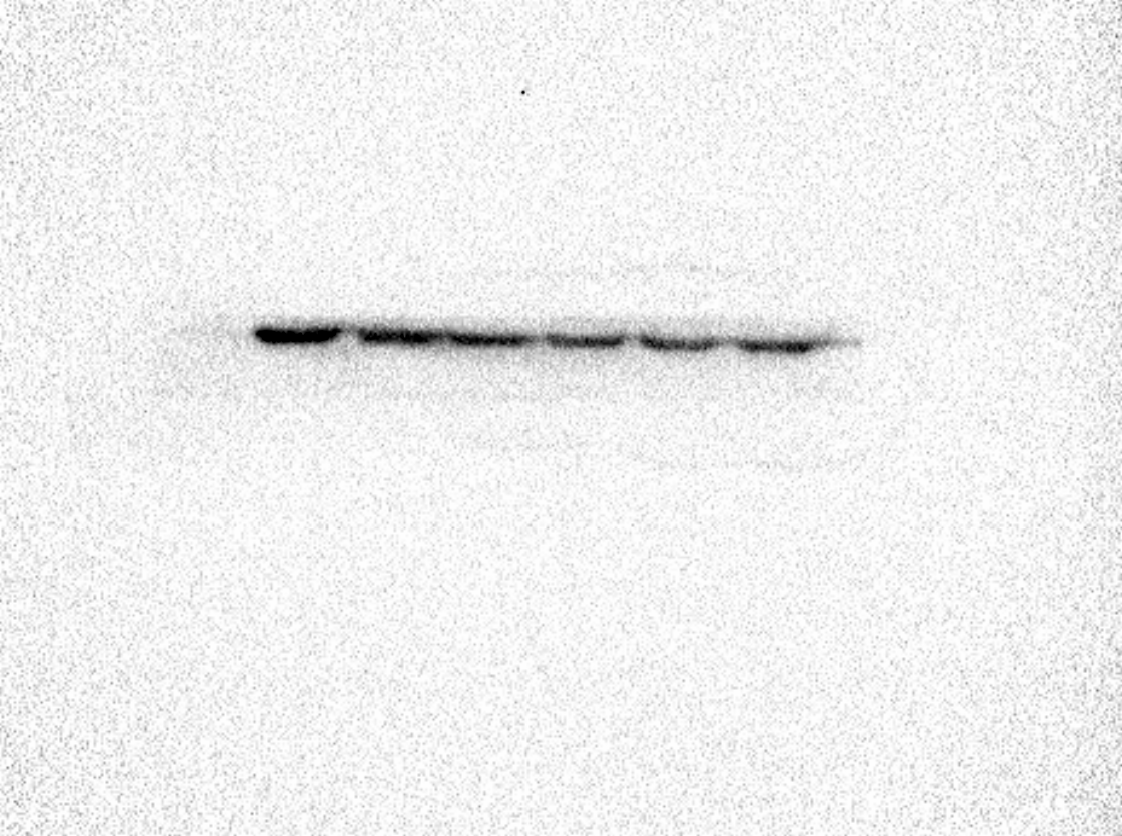

Supplement: Supplementary file 1 [file Data_Sheet_1.zip › Supplementary Data Sheets 4/Figure S5.tif]

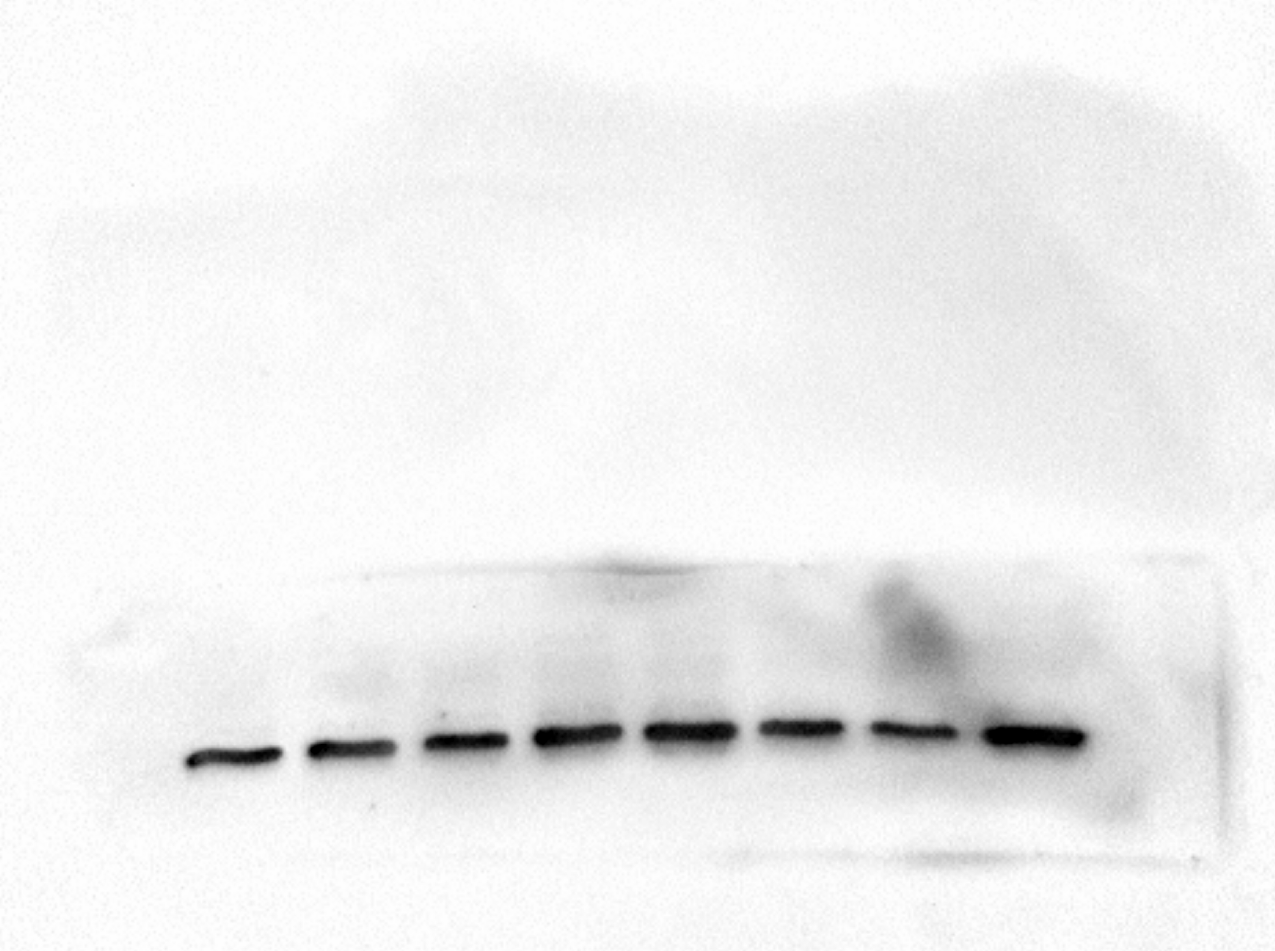

Supplement: Supplementary file 1 [file Data_Sheet_1.zip › Supplementary Data Sheets 4/Figure S7.tif]

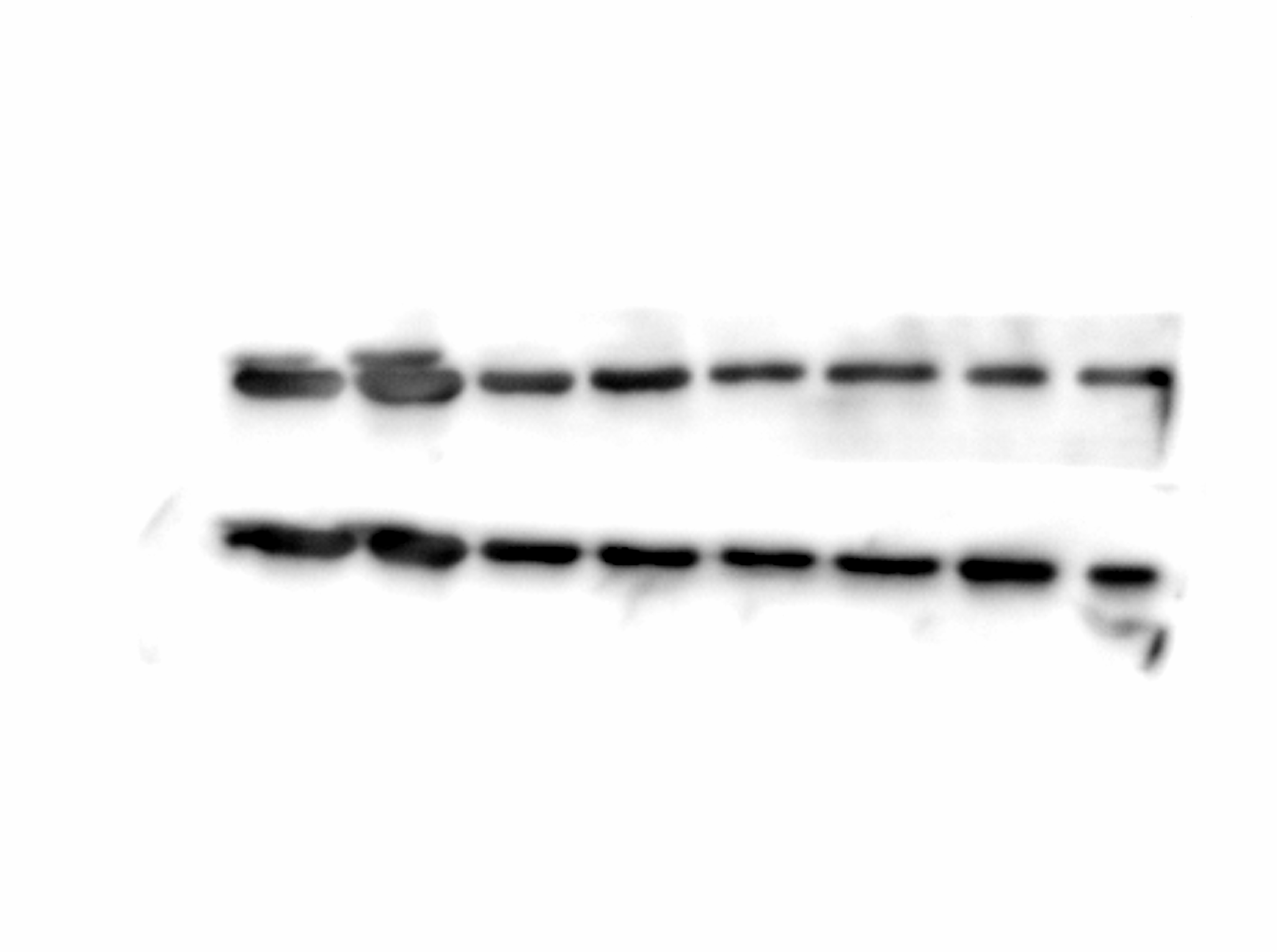

Supplement: Supplementary file 1 [file Data_Sheet_1.zip › Supplementary Data Sheets 4/Figure S8.tif]

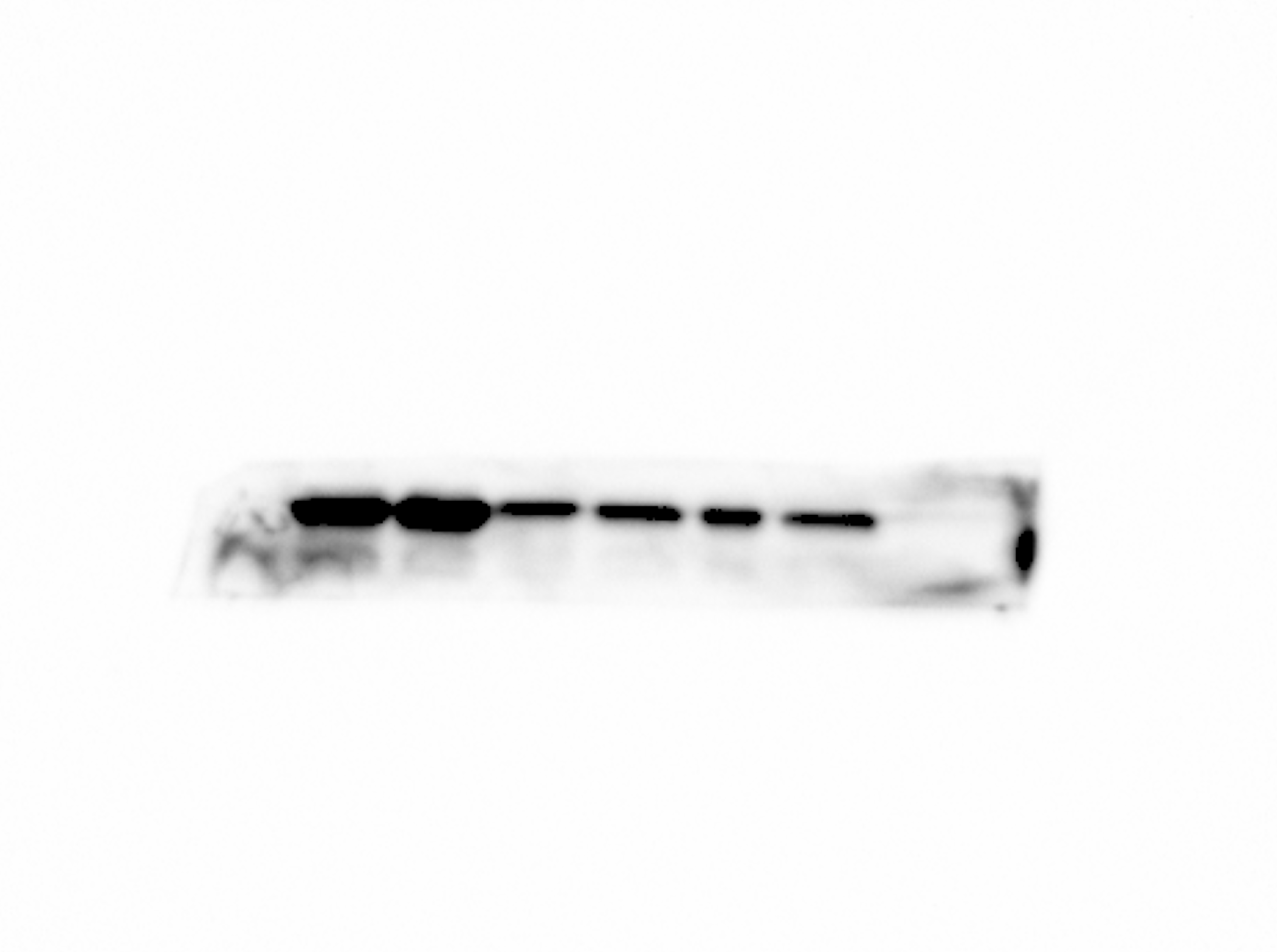

Supplement: Supplementary file 1 [file Data_Sheet_1.zip › Supplementary Data Sheets 4/Figure S9.tif]

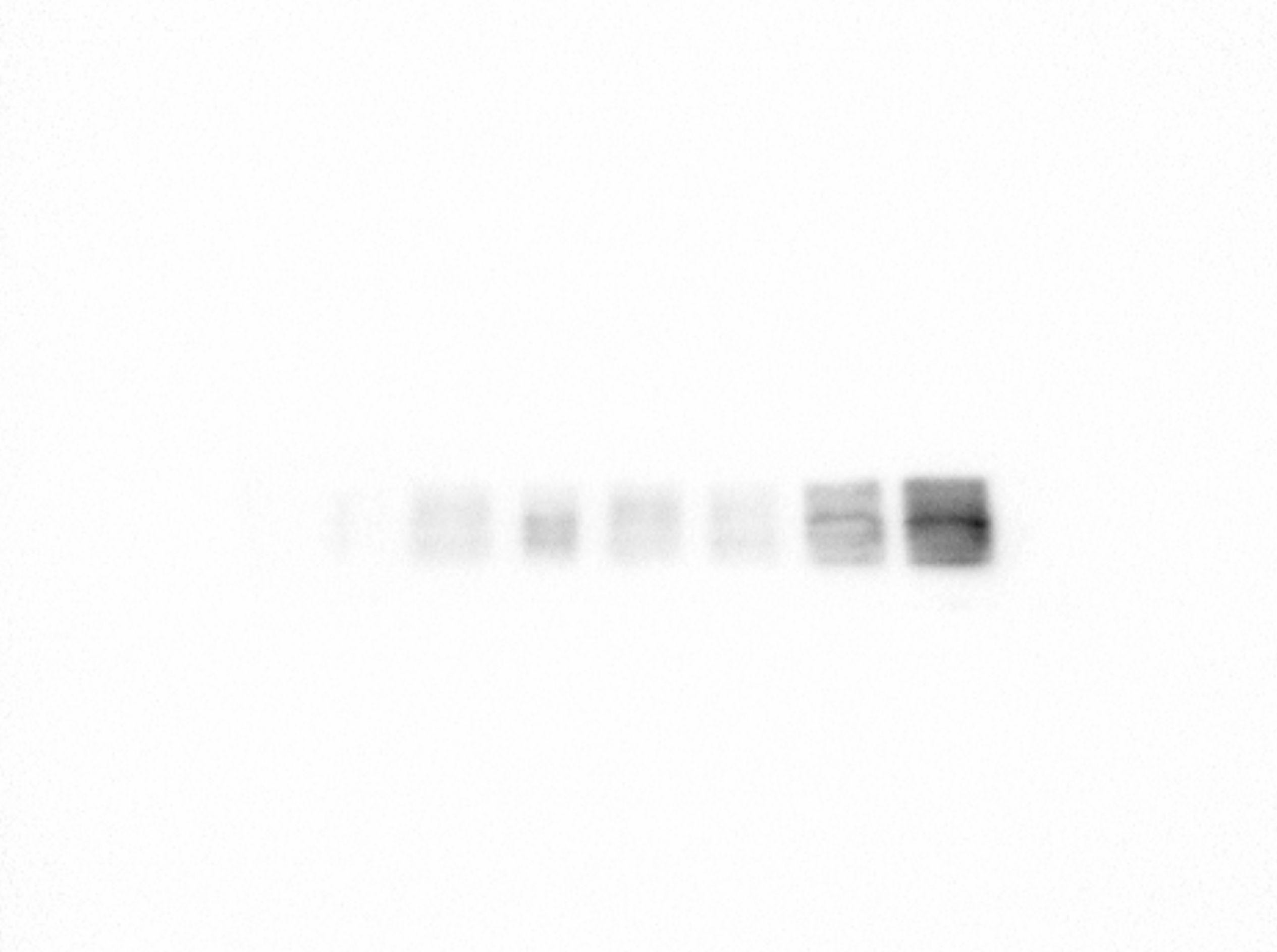

Supplement: Supplementary file 2 [file Data_Sheet_2.zip › Supplementary Data Sheets 5/Figure S11.tif]

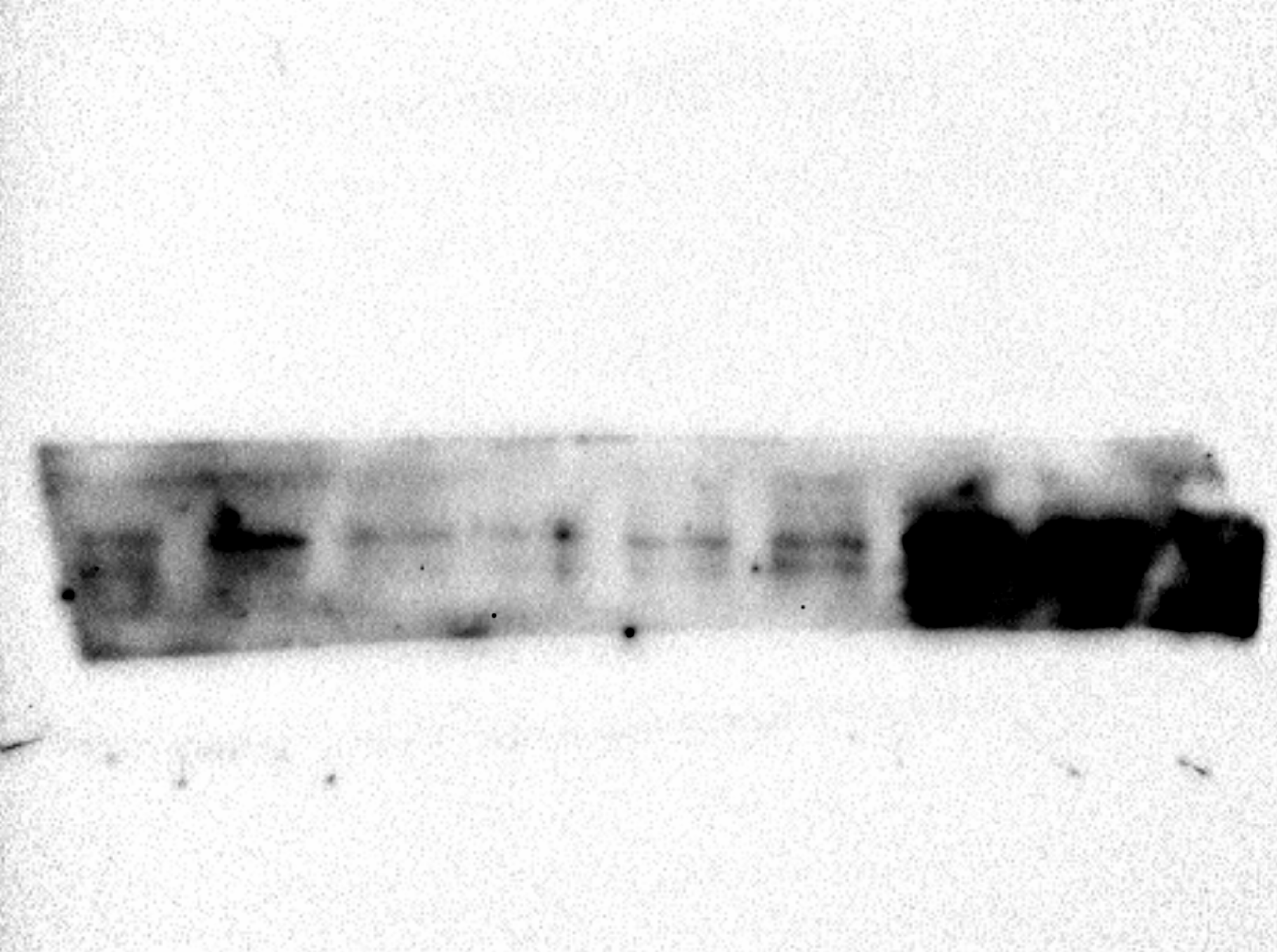

Supplement: Supplementary file 2 [file Data_Sheet_2.zip › Supplementary Data Sheets 5/Figure S12.tif]

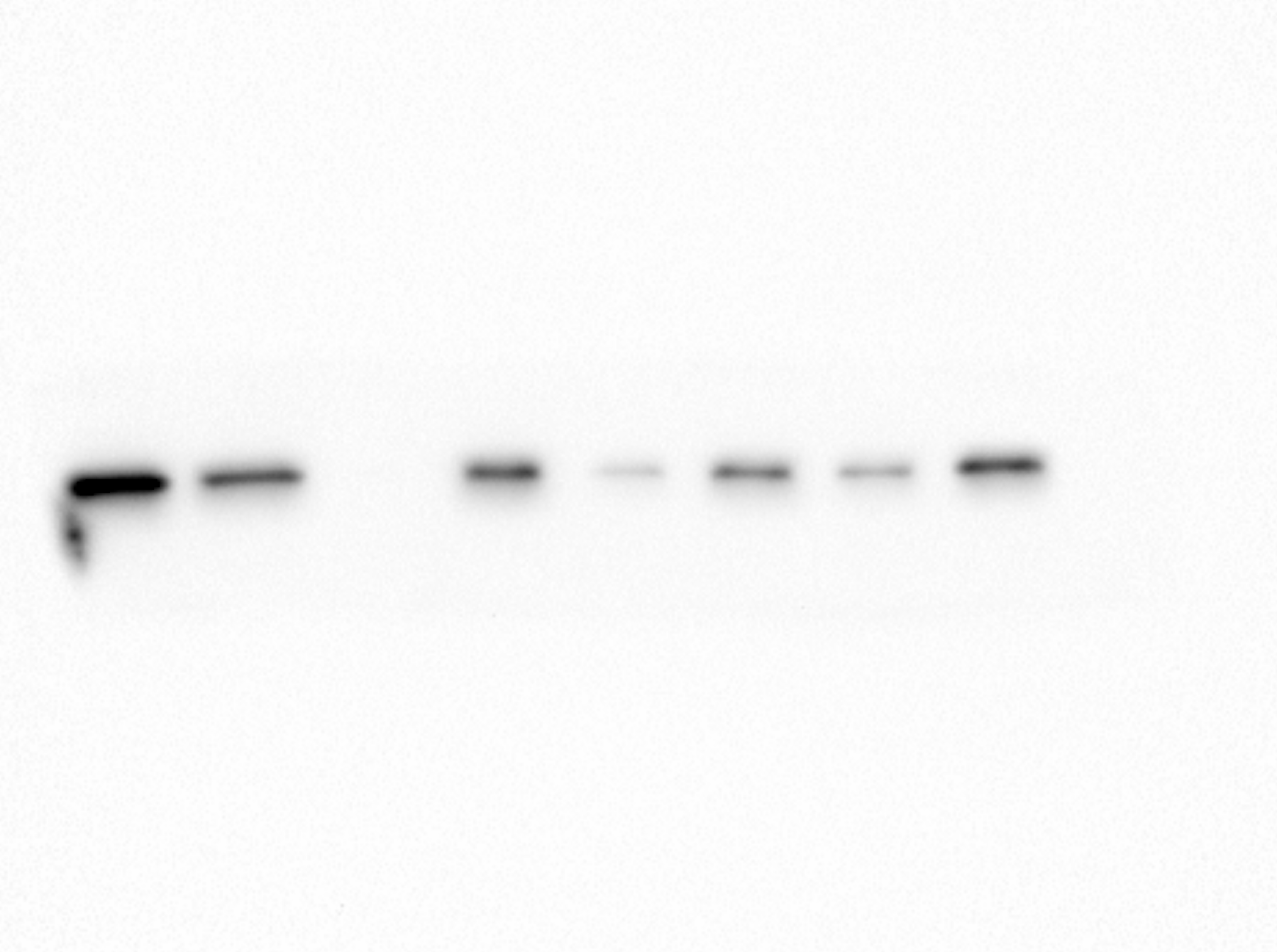

Supplement: Supplementary file 2 [file Data_Sheet_2.zip › Supplementary Data Sheets 5/Figure S13.tif]

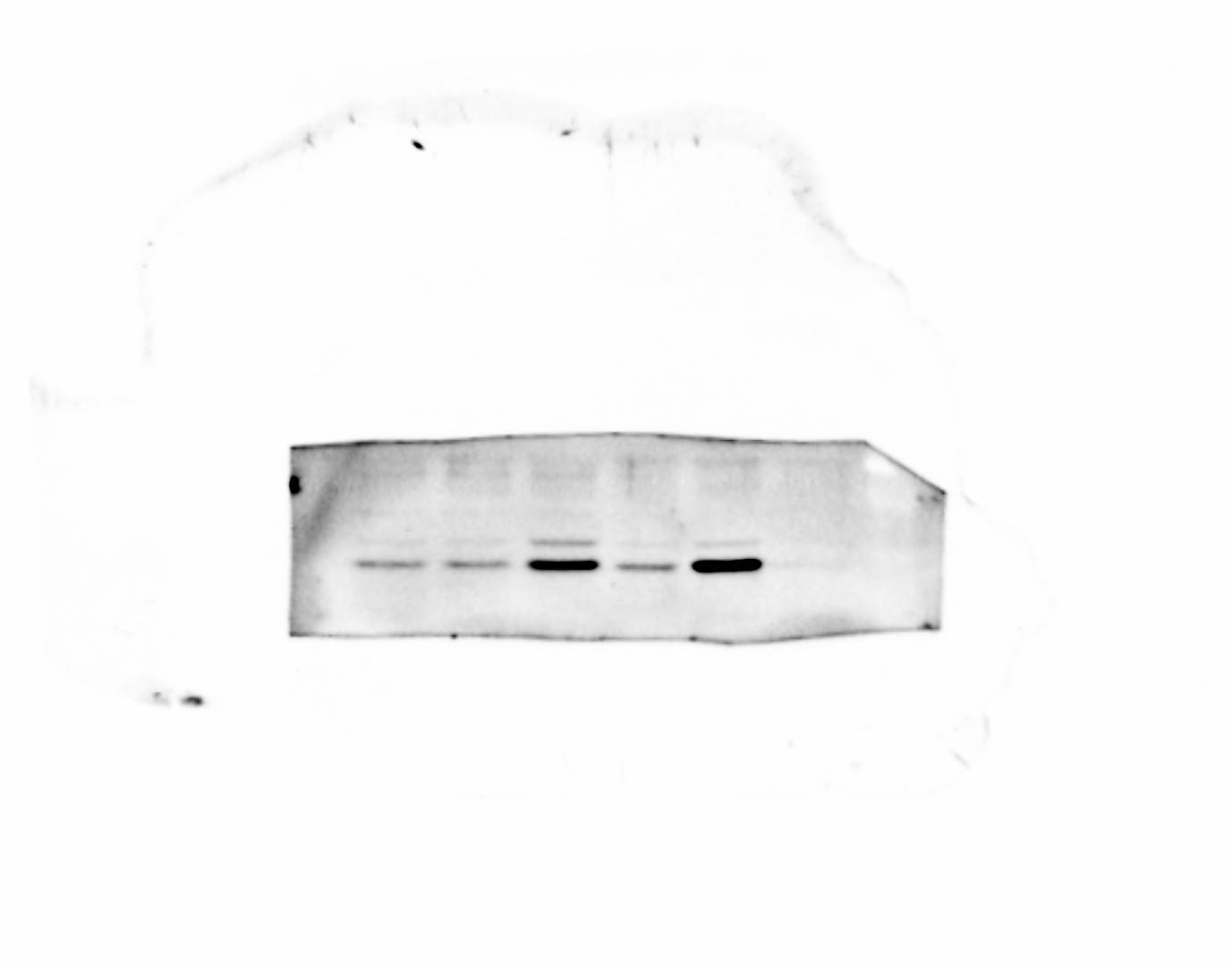

Supplement: Supplementary file 2 [file Data_Sheet_2.zip › Supplementary Data Sheets 5/Figure S14.tif]

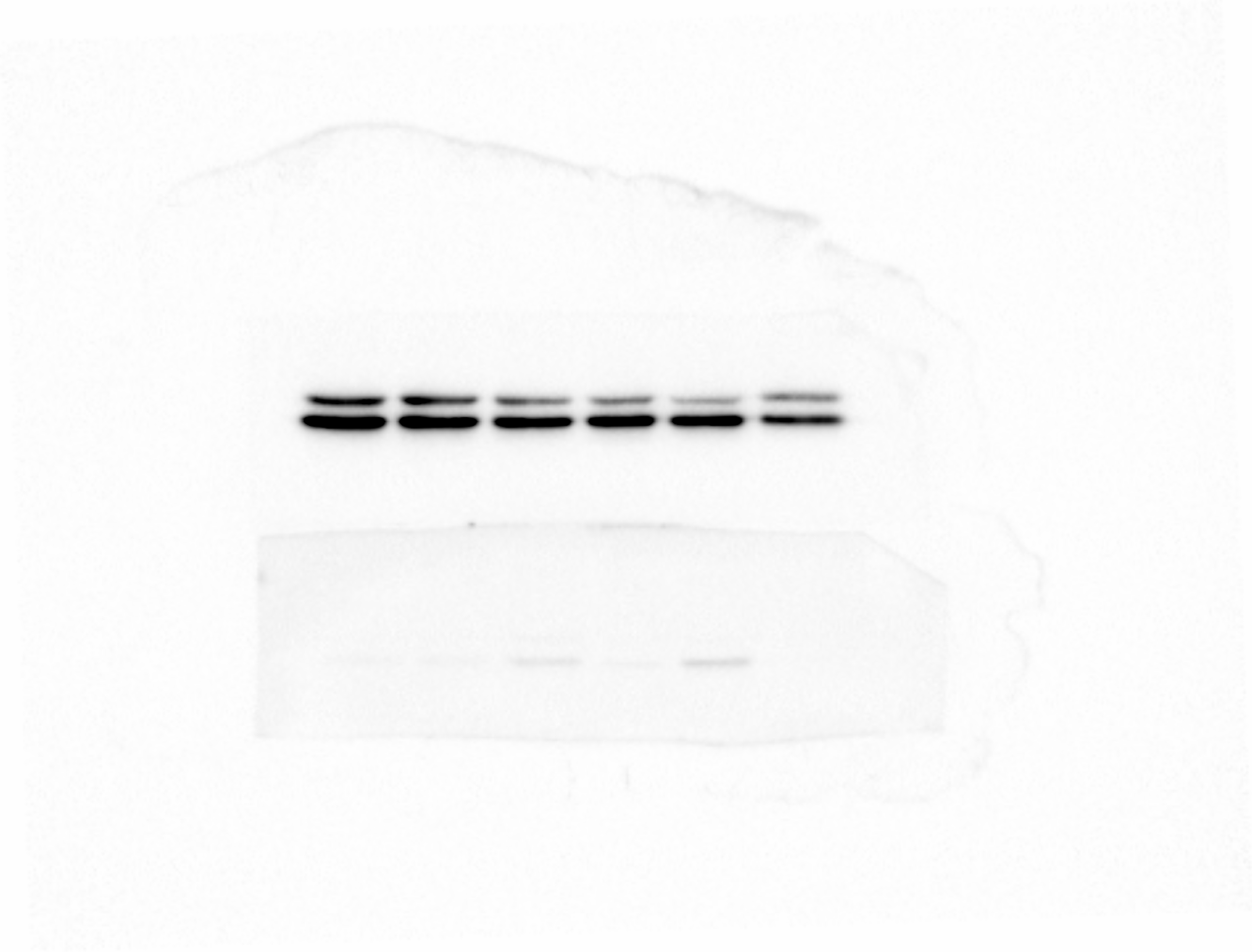

Supplement: Supplementary file 2 [file Data_Sheet_2.zip › Supplementary Data Sheets 5/Figure S15.tif]

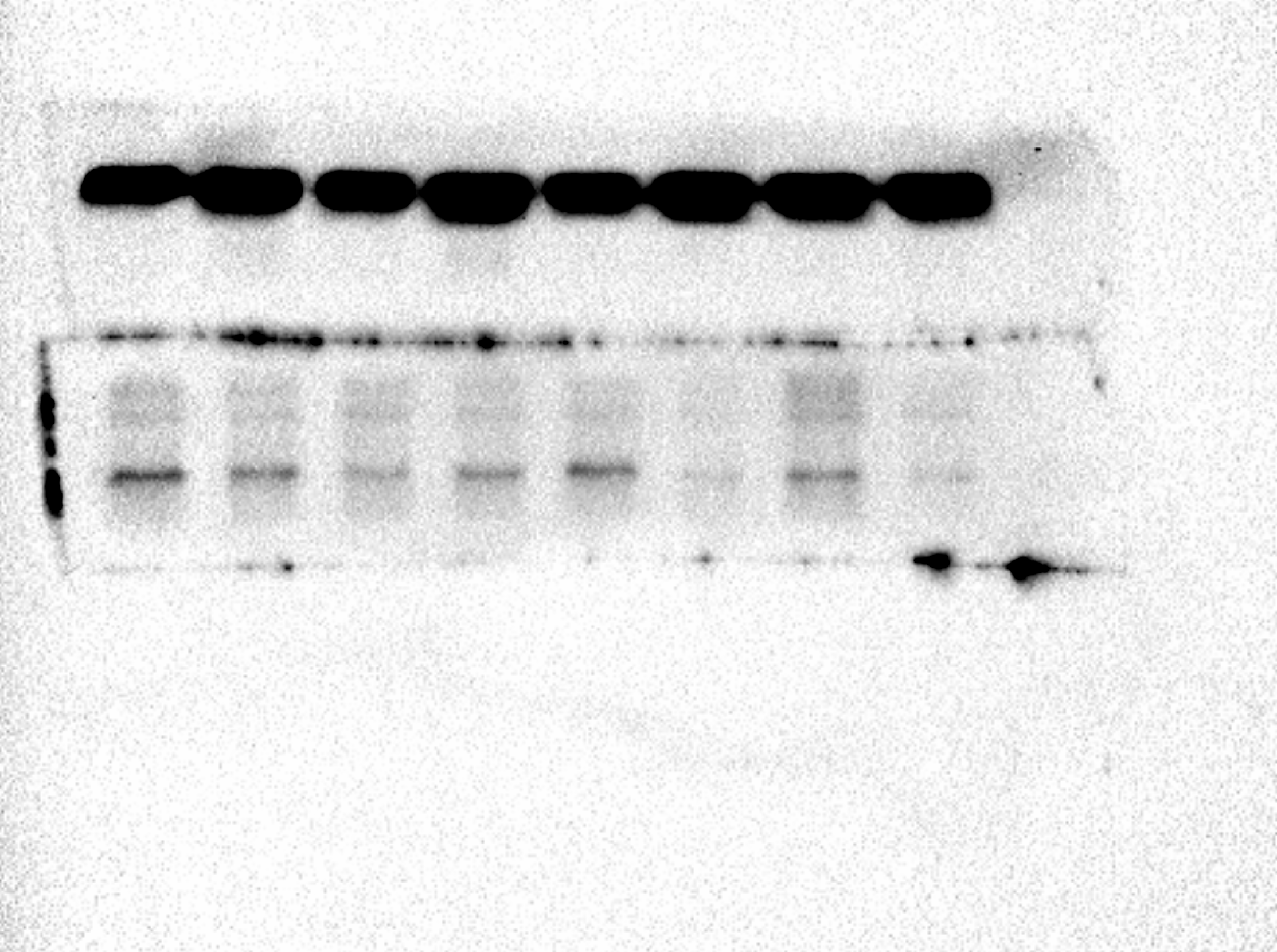

Supplement: Supplementary file 2 [file Data_Sheet_2.zip › Supplementary Data Sheets 5/Figure S16.tif]

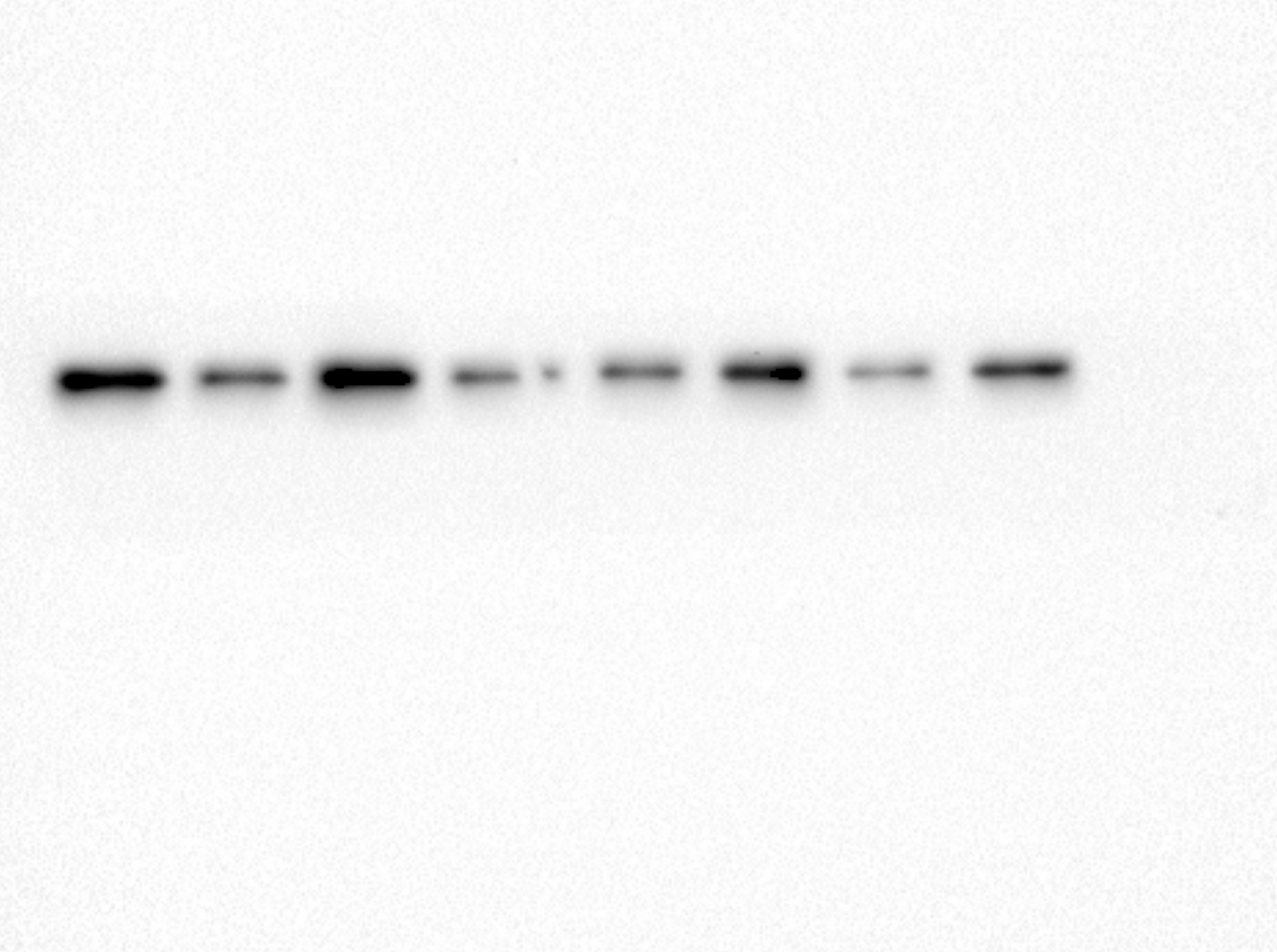

Supplement: Supplementary file 2 [file Data_Sheet_2.zip › Supplementary Data Sheets 5/Figure S17.tif]

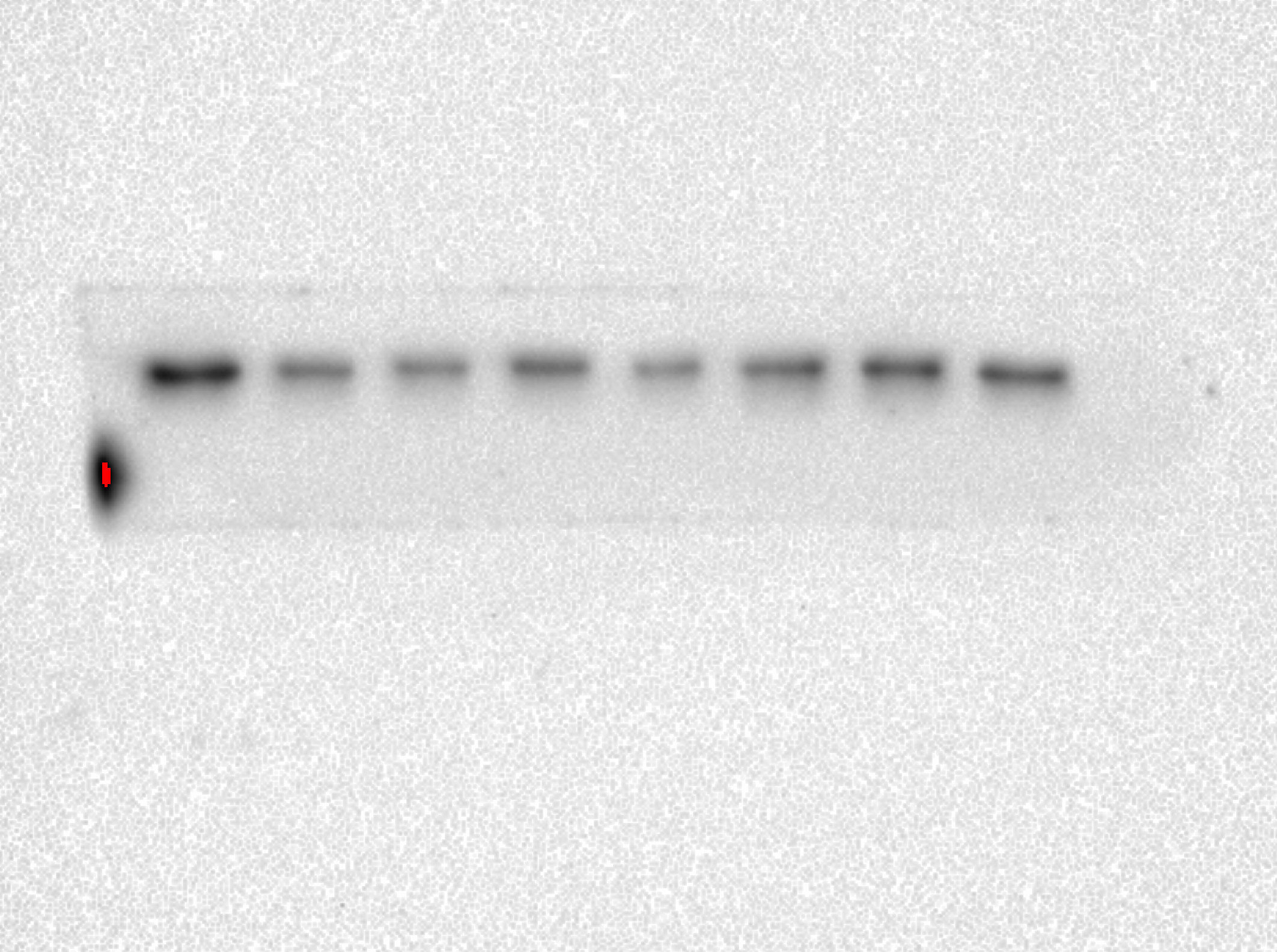

Supplement: Supplementary file 2 [file Data_Sheet_2.zip › Supplementary Data Sheets 5/Figure S18.tif]

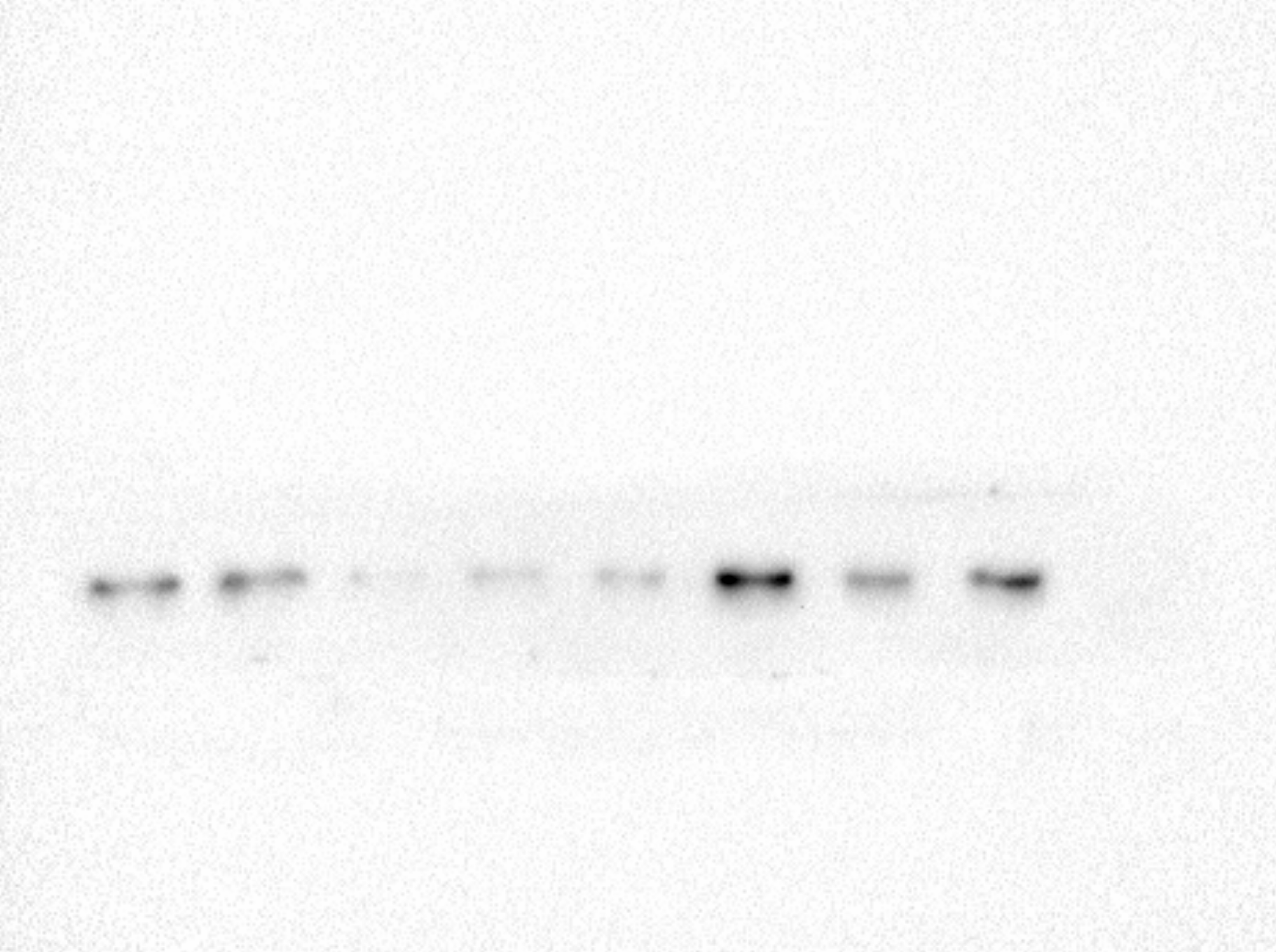

Supplement: Supplementary file 2 [file Data_Sheet_2.zip › Supplementary Data Sheets 5/Figure S19.tif]

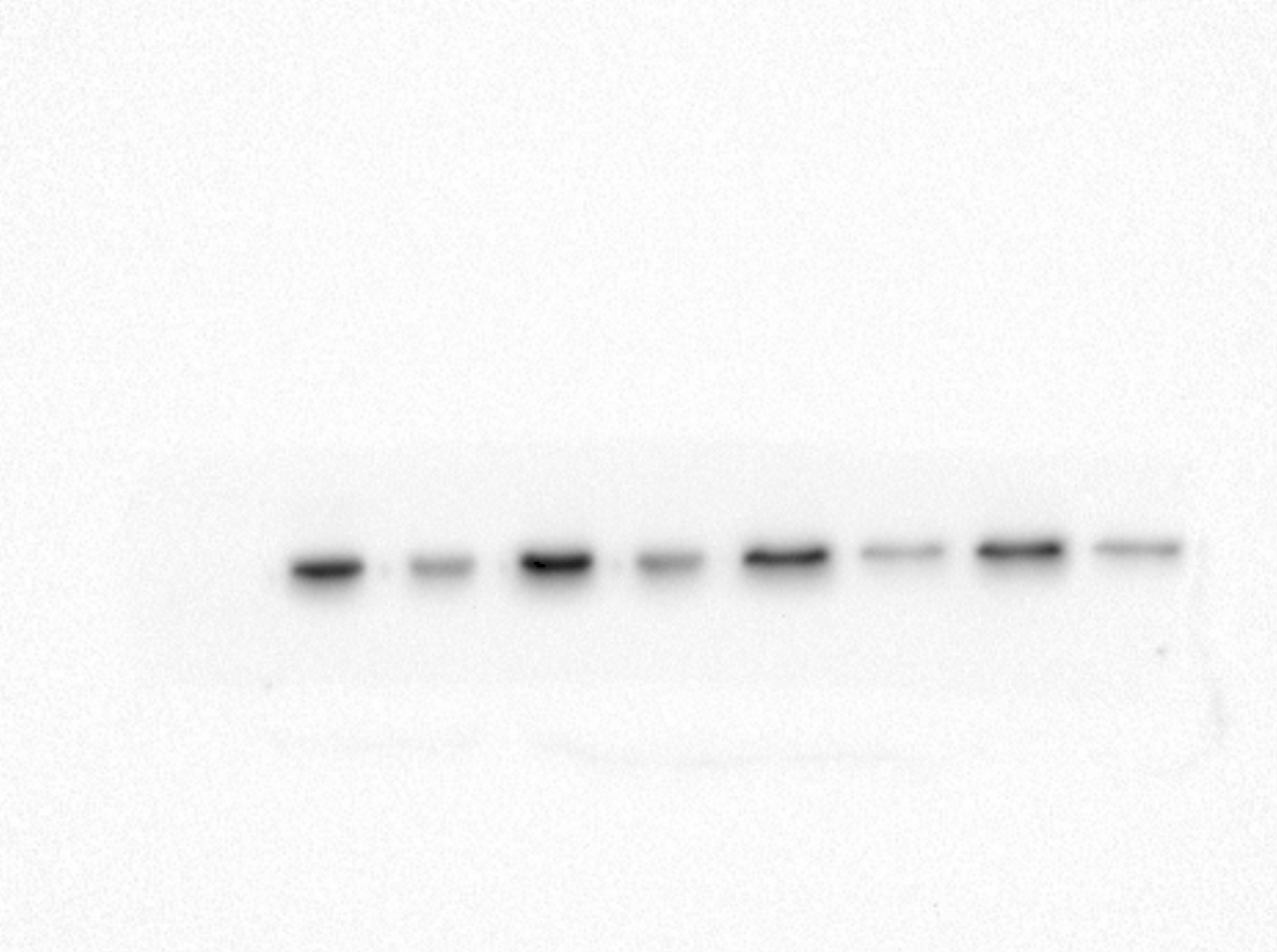

Supplement: Supplementary file 2 [file Data_Sheet_2.zip › Supplementary Data Sheets 5/Figure S20.tif]

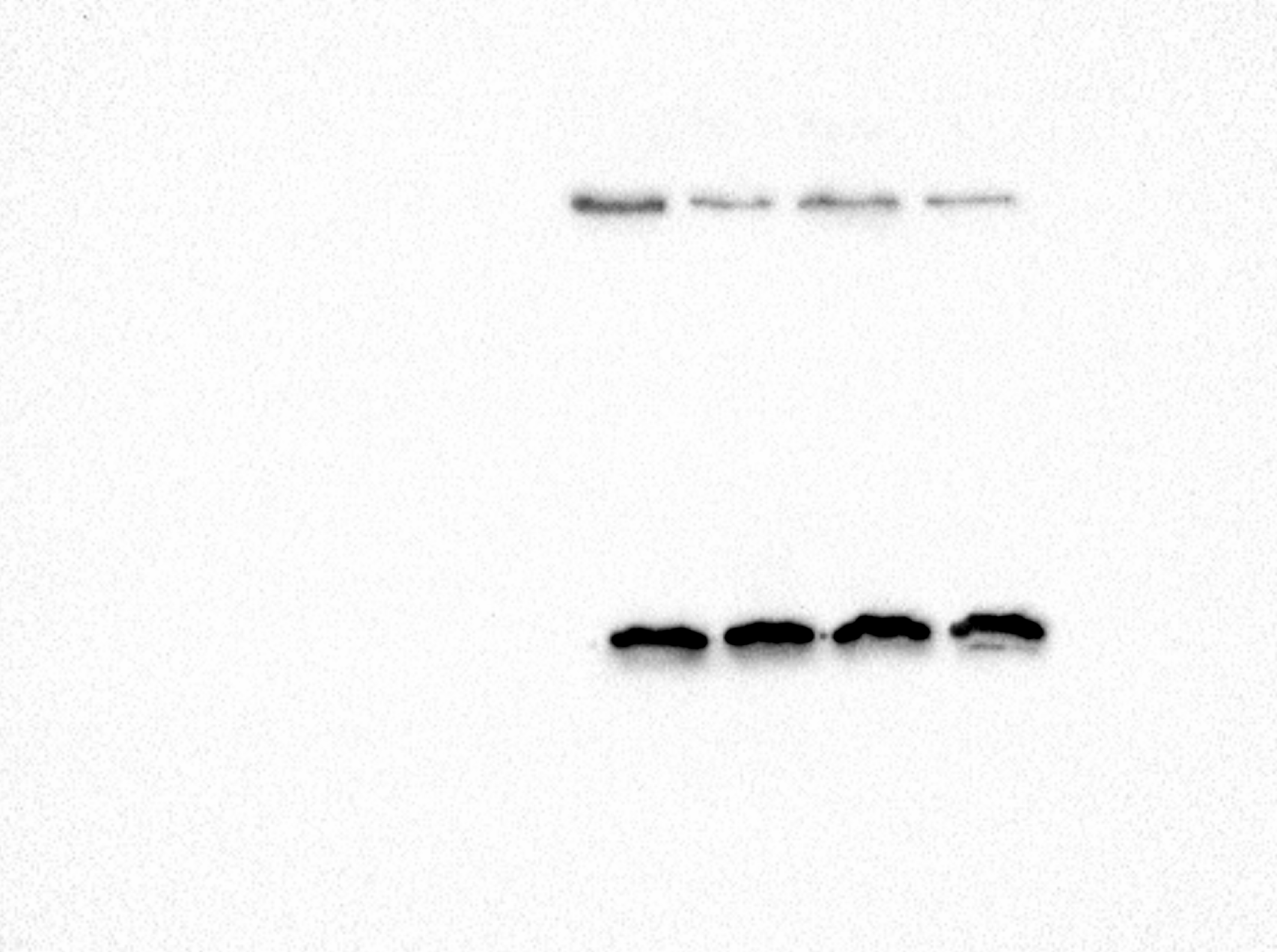

Supplement: Supplementary file 2 [file Data_Sheet_2.zip › Supplementary Data Sheets 5/Figure S21.tif]

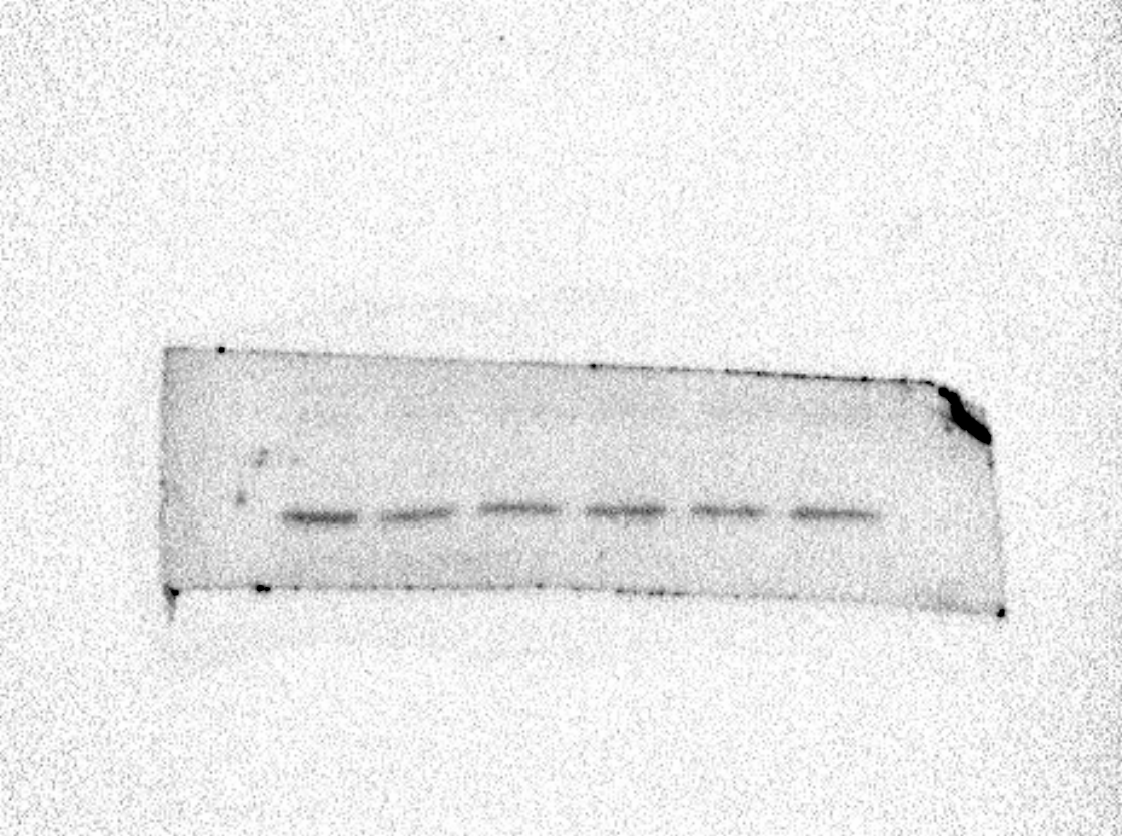

Supplement: Supplementary file 2 [file Data_Sheet_2.zip › Supplementary Data Sheets 5/Figure S22.tif]
