# Supplementary material for: PD-1-Mediated PI3K/Akt/mTOR, Caspase 9/Caspase 3 and ERK Pathways Are Involved in Regulating the Apoptosis and Proliferation of CD4+ and CD8+ T Cells During BVDV Infection in vitro
Source: Front Immunol. 2020 Mar 17;11:467. doi: 10.3389/fimmu.2020.00467 (PMC7089960; doi:10.3389/fimmu.2020.00467)
Supplement: Supplementary file 1 [file Data_Sheet_1.zip › Supplementary Data Sheets 4/Additional file 1.doc]

**Supplementary Figure Legends**

**Figure S1.** Western blot analysis of Akt in in BVDV-infected CD4^+^ and CD8^+^ T cells.

**Figure S2.** Western blot analysis of ERK in in BVDV-infected CD4^+^ and CD8^+^ T cells.

**Figure S3.** Western blot analysis of mTOR in in BVDV-infected CD4^+^ and CD8^+^ T cells.

**Figure S4.** Western blot analysis of PI3K in in CP BVDV-infected CD4^+^ and CD8^+^ T cells.

**Figure S5.** Western blot analysis of PI3K in in NCP BVDV-infected CD4^+^ and CD8^+^ T cells.

**Figure S6.** Western blot analysis of PI3K in in NCP BVDV-infected CD4^+^ and CD8^+^ T cells.

**Figure S7.** Western blot analysis of β-actin in NCP BVDV-infected CD4^+^ T cells.

**Figure S8.** Western blot analysis of β-actin in CP BVDV-infected CD4^+^ T cells.

**Figure S9.** Western blot analysis of β-actin in NCP BVDV-infected CD8^+^ T cells.

**Figure S10.** Western blot analysis of β-actin in BVDV-infected CD21^+^ B cells.

**Figure S11.** Western blot analysis of p-Akt in CP BVDV-infected CD4^+^ T cells.

**Figure S12.** Western blot analysis of p-Akt in CP BVDV-infected CD8^+^ T cells.

**Figure S13.** Western blot analysis of p-Akt in NCP BVDV-infected CD4^+^ and CD8^+^ T cells.

**Figure S14.** Western blot analysis of p-ERK in CP BVDV-infected CD4^+^ and CD8^+^ T cells.

**Figure S15.** Western blot analysis of p-ERK in NCP BVDV-infected CD4^+^ and CD8^+^ T cells.

**Figure S16.** Western blot analysis of p-mTOR in NCP BVDV-infected CD8^+^ T cells.

**Figure S17.** Western blot analysis of p-mTOR in CP BVDV-infected CD4^+^ T cells.

**Figure S18.** Western blot analysis of p-PI3K in NCP BVDV-infected CD4^+^ T cells.

**Figure S19.** Western blot analysis of p-PI3K in NCP BVDV-infected CD8^+^ T cells.

**Figure S20.** Western blot analysis of PD-1 in NCP BVDV-infected CD4^+^ and CD8^+^ T cells.

**Figure S21.** Western blot analysis of PD-1 in CP BVDV-infected CD8^+^ T cells.

**Figure S22.** Western blot analysis of PD-1 in BVDV-infected CD21^+^ B cells.
